# Synthesis and therapeutic potential of imidazole containing compounds

**DOI:** 10.1186/s13065-020-00730-1

**Published:** 2021-02-18

**Authors:** Ankit Siwach, Prabhakar Kumar Verma

**Affiliations:** Department of Pharmaceutical Sciences, Maharshi Dayanand University, Rohtak, Haryana India

**Keywords:** 1, 3-diazole, Antibacterial, Antitumor, Antioxidant, Antitubercular

## Abstract

Imidazole is a five-membered heterocyclic moiety that possesses three carbon, two nitrogen, four hydrogen atoms, and two double bonds. It is also known as 1, 3-diazole. It contains two nitrogen atoms, in which one nitrogen bear a hydrogen atom, and the other is called pyrrole type nitrogen. The imidazole name was reported by Arthur Rudolf Hantzsch (1857–1935) in 1887. 1, 3-diazole is an amphoteric in nature i.e. it shows both acidic and basic properties. It is a white or colorless solid that is highly soluble in water and other polar solvents. Due to the presence of a positive charge on either of two nitrogen atom, it shows two equivalent tautomeric forms. Imidazole was first named glyoxaline because the first synthesis has been made by glyoxal and ammonia. It is the basic core of some natural products such as histidine, purine, histamine and DNA based structures, etc. Among the different heterocyclic compounds, imidazole is better known due to its broad range of chemical and biological properties. Imidazole has become an important synthon in the development of new drugs. The derivatives of 1, 3-diazole show different biological activities such as antibacterial, antimycobacterial, anti-inflammatory, antitumor, antidiabetic, anti-allergic, antipyretic, antiviral, antioxidant, anti-amoebic, antihelmintic, antifungal and ulcerogenic activities, etc. as reported in the literature. There are different examples of commercially available drugs in the market which contains 1, 3-diazole ring such as clemizole (antihistaminic agent), etonitazene (analgesic), enviroxime (antiviral), astemizole (antihistaminic agent), omeprazole, pantoprazole (antiulcer), thiabendazole (antihelmintic), nocodazole (antinematodal), metronidazole, nitroso-imidazole (bactericidal), megazol (trypanocidal), azathioprine (anti rheumatoid arthritis), dacarbazine (Hodgkin's disease), tinidazole, ornidazole (antiprotozoal and antibacterial), etc. This present review summarized some pharmacological activities and various kinds of synthetic routes for imidazole and their derived products. 
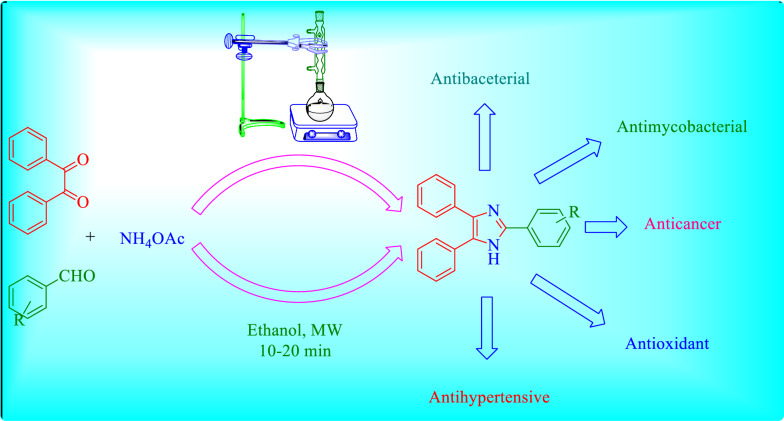

## Background

Nowadays, Public health problems were increasing due to AMR in drug therapy. So, there is necessary for the development of a new drug that overcomes the AMR problems [1].

In past, those drugs which contain heterocyclic nuclei give high chemotherapeutic values and act as a remedy for the development of novel drugs [2]. There are lots of heterocyclic compounds that are in clinical use to treat infectious diseases. So, there is a great importance of heterocyclic ring containing drugs [3].

**Fig. 1:**
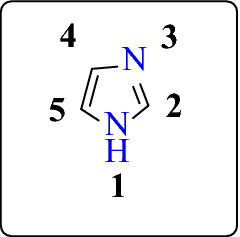
Imidazole

In heterocyclic chemistry, imidazole containing moiety occupied a unique position [4]. It is a five-membered nitrogenous heterocyclic moiety that possesses three carbon, two nitrogen, four hydrogen atoms, and two double bonds having general molecular formula is C_3_H_4_N_2_ (Fig. 1). The nitrogen atoms present at the first and third positions (non–adjacent position) of the ring [5], position four and five are equivalent [6]. It is also known as 1,3-diazole. It contains two nitrogen atoms, one nitrogen bear a hydrogen atom, and the other is called pyrrole type nitrogen [7]. 1,3-diazole ring is a bioester of the pyrazole ring [8]. It is the basic core of some natural products such as histidine, purine, histamine and DNA based structures, etc. [4]. The imidazole name was first reported by Arthur Rudolf Hantzsch (1857–1935) in 1887 [6].

1,3-diazole shows an amphoteric phenomenon i.e. it can behave like acid as well as a base. Two types of lone pair are present in the imidazole ring, delocalized and non-delocalized (non-Huckle) lone pair, i.e. both nitrogen of 1,3-diazole shows different dissociation constant. The dissociation constant (pKa) of delocalized lone pair and non-delocalized lone pair is 7 and 14.9 respectively. 1,3-diazole ring is susceptible to both electrophilic and nucleophilic attacks due to its amphoteric phenomenon [7]**.** For an acid imidazole, the dissociation constant is 14.5, which makes it less acidic than phenol, imides, and carboxylic acid except for alcohols (which is less acidic than imidazole). For a basic imidazole, the dissociation constant (pKa) is approximately 7 (which makes imidazole 60 times more basic than pyridine). The acidic proton is present on the first nitrogen atom of the imidazole ring [6].

**Fig. 2:**
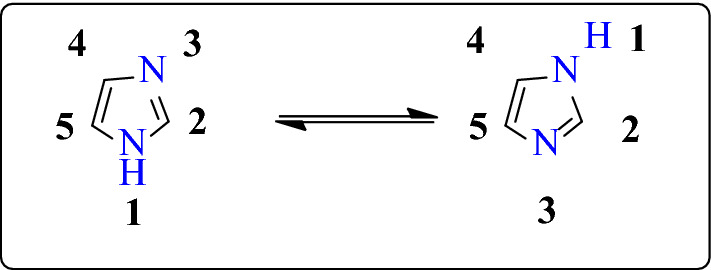
Tautomeric forms of imidazole

Due to the presence of a positive charge on either of the two nitrogen atoms, 1,3-diazole ring shows two equivalent tautomeric forms (Fig. 2) [9]. The presence of a sextet of π-electrons on the ring makes it an aromatic compound. The nitrogen atom on the third position in the imidazole ring is more reactive to the electrophilic compound due to the availability of unshared pairs of electron on the second nitrogen atom since the second nitrogen is a part of aromatic sextet [6].

It is a white or colorless solid. The imidazole ring shows excellent solubility in water and other polar solvents [10]. The dipole moment, melting point, and boiling point of the imidazole ring is 4.8 D in dioxane [6], 88.9^ °^C, and 267.8^ °^C [7] respectively. It possesses intramolecular hydrogen bonding [9].

Imidazole was first named glyoxaline because the first synthesis has been made by glyoxal and ammonia [9]. There is a different kind of synthetic route from which we can synthesize 1,3-diazloes, and its derivatives. Common methods are Debus-Radiszewski synthesis, Wallach synthesis, from dehydrogenation of imidazolines, from alpha halo-ketones, Marckwald synthesis, and amino nitrile [11].

Due to the polar nature of the imidazole ring, the pharmacokinetic parameters of the imidazole containing compounds should be improved to a great extent. Thus, this moiety helps to overcome the solubility problems of poorly soluble drug entities [12].

The 1,3-diazole and it's containing compounds shows a lot of therapeutic activities such as analgesics, antifungal, antihypertensive, antiobesity, antitumor [3], antiviral, anthelmintic, antitubercular [4], antiulcer, antihistaminic [13], anti-inflammatory, antidepressant [14]. antidiabetic [15], anticonvulsant [16], antiallergic [7], antirheumatic [17], antiasthmatic, alpha-blockers [18], antiprotozoal [19], antiaging, anticoagulant, antimalarial [20], and antiamoebic activity [21] etc.

There are different examples of commercially available drugs which consist 1,3,4-oxadiazole ring (Table 1) such as clemizole (antihistaminic agent), etonitazene (analgesic), enviroxime (antiviral), irtemazole, astemizole (antihistamine), omeprazole, pantoprazole (antiulcer), thiabendazole (antihelmintic), nocodazole (antinematodal) [22], metronidazole and nitrosoimidazole (bactericidal), megazol (trypanocidal) [12], azathioprine (anti-rheumatoid arthritis), tinidazole, ornidazole (antiprotozoal and antibacterial), satranidazole (amoebiasis), cimetidine (gastric ulcer), carbimazole (against thyroid disorder), tolazoline (vasodilator action), naphazoline (vasoconstrictor), tetrahydrozoline (vasoconstrictor) [16], etomidate, lansoprazole, flumazenil, methimazole, pilocarpine [19], ketoconazole [23], dacarbazine (anticancer) [24], pimobendan (calcium sensitizer and phosphodiesterase inhibitor) [25], fenbendazole [26].

**Table 1:**
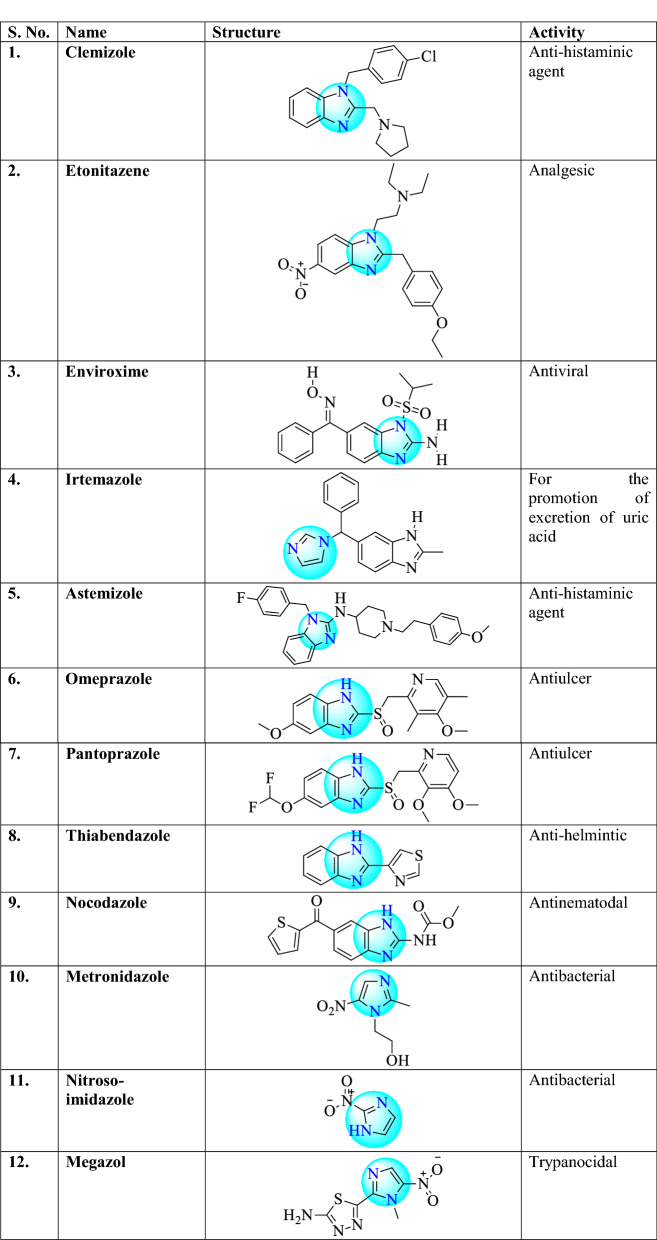

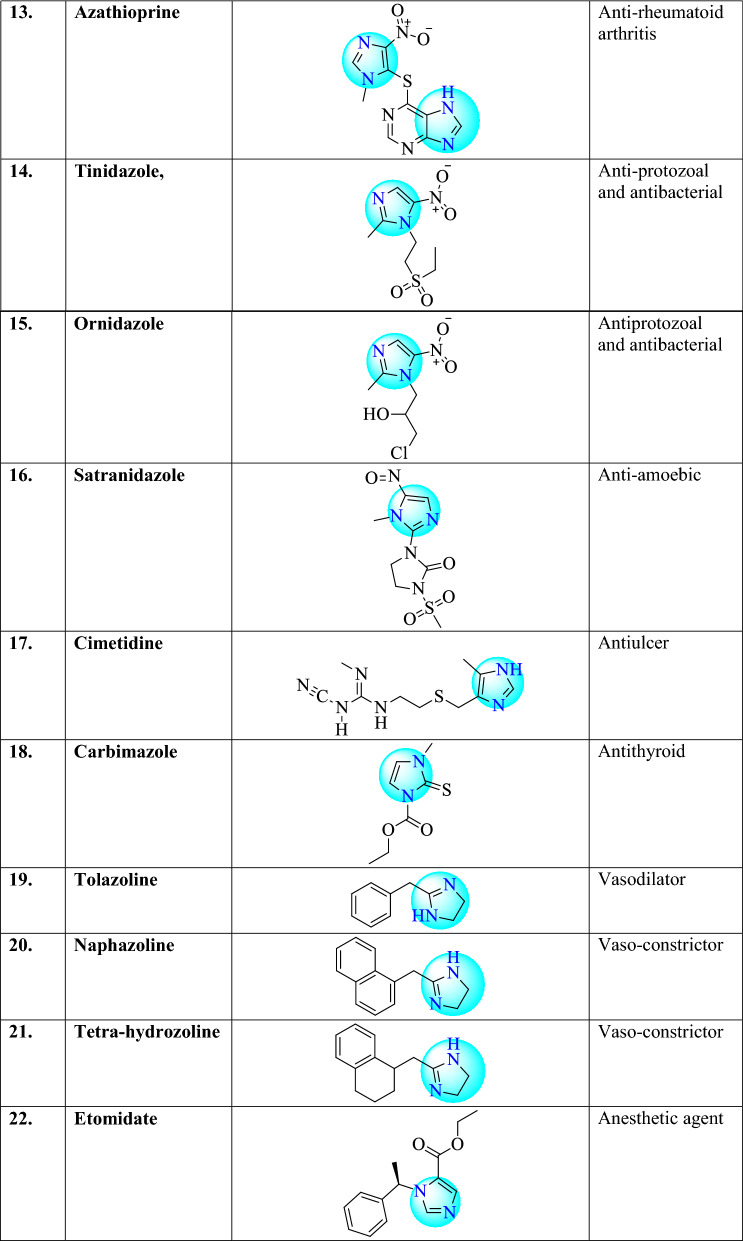

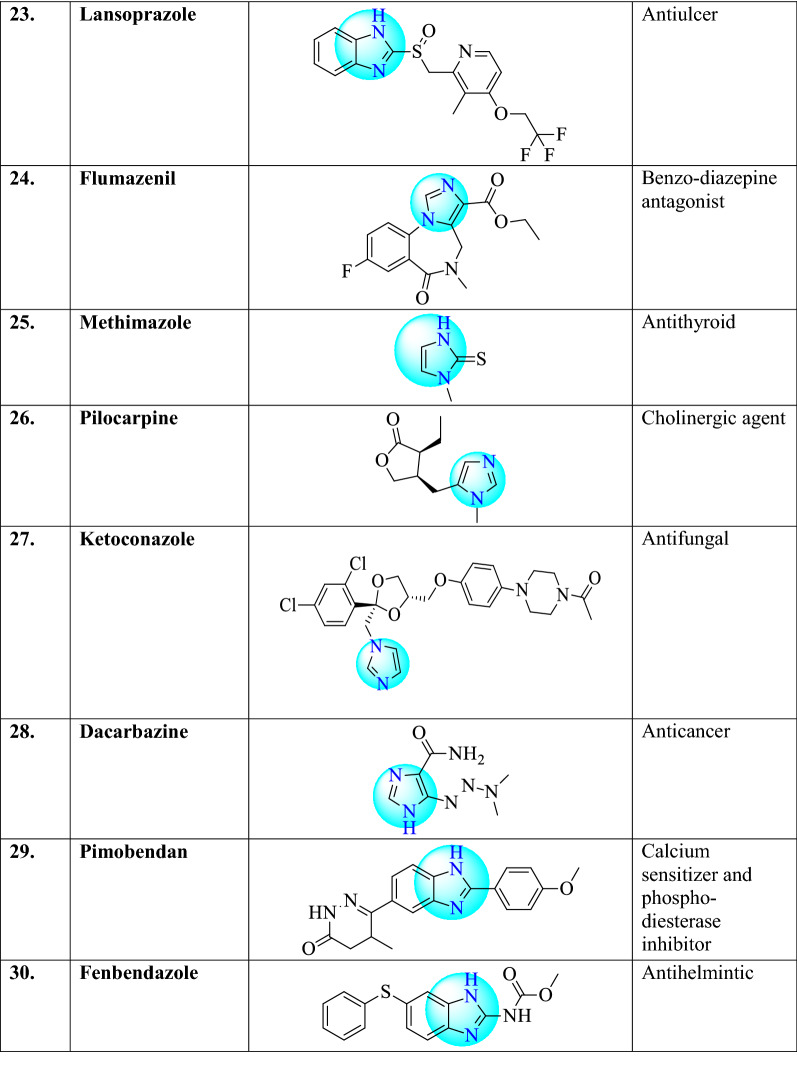
Commercially available drugs are containing Imidazole nucleus

### The mechanism for the formation of 2,4,5-trisubstituted imidazole

**Scheme 1:**
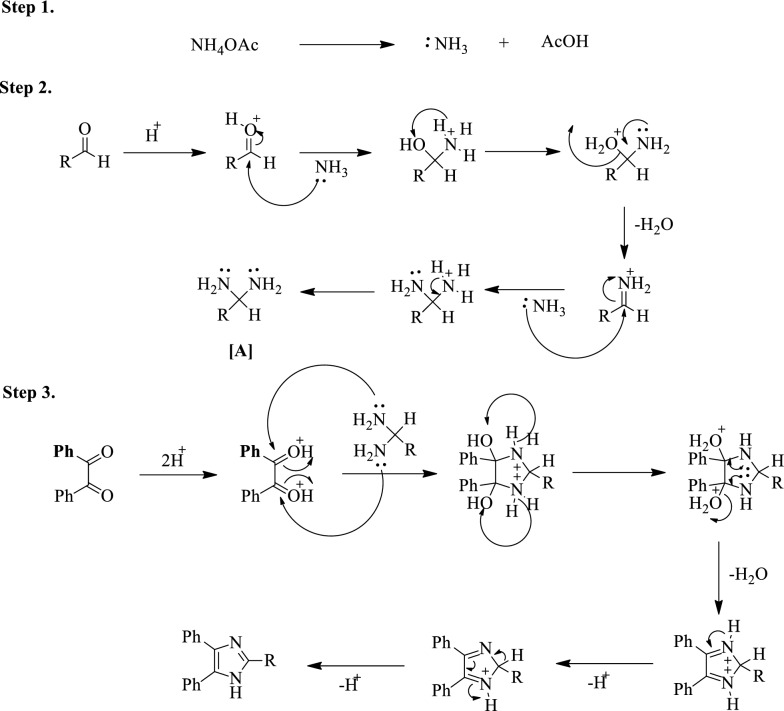
Plausible mechanism for the synthesis of imidazoles catalyzed by (4–SB)T(4–SPh)PHSO_4_

The Debus-Radziszewski reaction mechanism for the formation of the 2,4,5-trisubstituted imidazole is given by (Scheme 1) [27].

## Main text

### Antibacterial activity

**Scheme 2:**
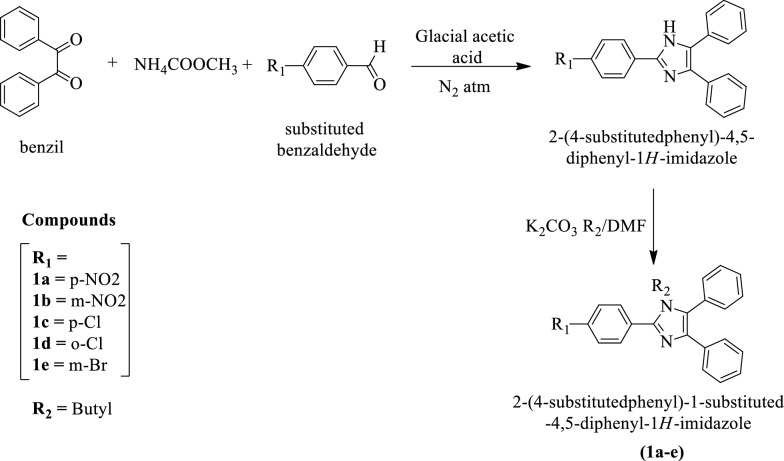
Synthesis of 2-(4-substitutedphenyl)-1-substituted-4,5-diphenyl-1*H-*imidazole

Jain et al. [28] synthesized 2-(4-substituted phenyl)-1-substituted-4, 5-diphenyl-1H-imidazole (Scheme 2) and evaluated their antimicrobial activity against *S. aureus, E. coli*, and *B. subtilis* by cylinder wells diffusion method using Norfloxacin as a reference drug. Among the different derivatives, compounds **1a** and **1b** showed good antimicrobial potential. The conclusion of antibacterial activity was presented in (Table 2, Jain et al. [28]).

**Table 2:** Antibacterial activity of synthesized derivatives (1a-e)-zone of inhibition (mm,%) Jain et al. [28]

Compounds	Zone of inhibition					
	*S. aureus*		*B. subtilis*		*E. coli*	
	50(µg/mL)	150(µg/mL)	50(µg/mL)	150(µg/mL)	50(µg/mL)	150(µg/mL)
1a	5 (23.09)	9 (42.85)	4 (19.04)	8 (38.09)	7 (33.33)	9 (42.85)
1b	3 (14.28)	7 (33.33)	4 (19.04)	7 (33.33)	6 (28.57)	9 (42.85)
1c	5 (23.09)	6 (28.57)	6 (28.57)	7 (33.33)	5 (23.09)	8 (38.09)
1d	5 (23.09)	6 (28.57)	6 (28.57)	6 (28.57)	5 (23.09)	8 (38.09)
1e	4 (19.04)	7 (33.33)	4 (19.04)	7 (33.33)	5 (23.09)	8 (38.09)
Norfloxacin*	21	–	21	–	21	–

Norfloxacin* Norfloxacin at concentration 50(µg/mL)

**Scheme 3:**
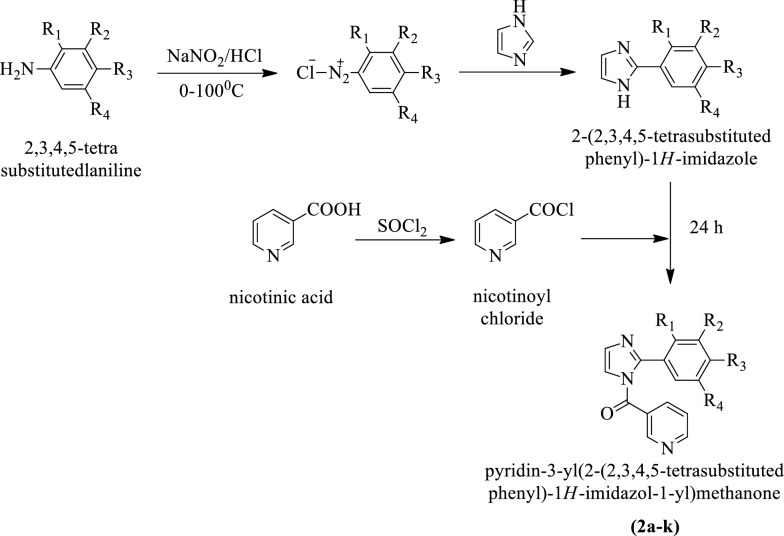
Synthesis of pyridin-3-yl(2-(2,3,4,5-tetrasubstitutedphenyl)-1*H-*imidazol-1-yl)methanone

Narasimhan et al. [1] synthesized pyridin-3-yl (2-(2,3,4,5-tetra substituted phenyl)-1H-imidazol-1-yl) methanone (Scheme 3). The tube dilution method was used for the determination of antimicrobial potential against *S. aureus, B. subtilis,* and *E. coli* using ciprofloxacin as a reference drug. The antifungal activity of these derivatives was also evaluated against *A. niger* and *C. albicans* using Fluconazole as a reference standard. The conclusion of antimicrobial potential was presented in (Table 3, Narasimhan et al. [1]).

**Table 3:** Antimicrobial activity of titled compounds (2a-k) Narasimhan et al. [1]

Compounds	MIC (µM/mL)					
	*S. aureus*	*B. subtilis*	*E. coli*	*C. albicans*		*A. niger*
2a	0.012	0.003	0.003		0.025	0.050
2b	ND	ND	ND		0.022	0.005
2c	ND	ND	ND		0.005	0.005
2d	0.044	0.044	0.044		0.022	0.044
2e	0.022	0.044	0.006		0.022	0.044
2f	0.044	0.044	0.011		0.342	0.044
2g	ND	ND	ND		0.004	0.019
2h	0.010	0.010	0.040		0.020	0.040
2i	0.040	0.002	0.040		0.020	0.040
2j	0.013	0.005	0.002		0.025	0.025
2k	0.002	0.002	0.002		0.020	0.040
Ciprofloxacin	0.004	0.004	0.004		–	–
Fluconazole	–	–	–		0.005	0.005

*MIC* Minimum inhibitory concentration, *ND* not detected

**Scheme 4:**
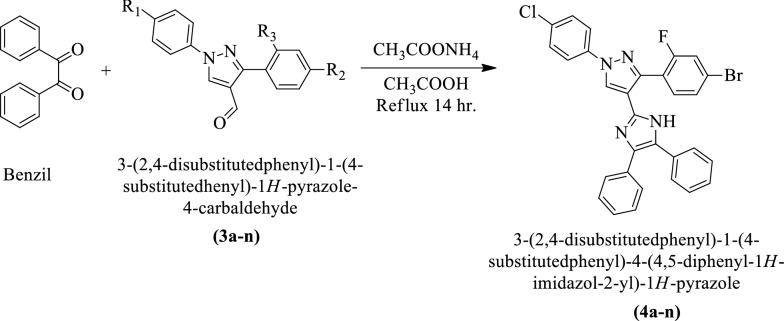
Synthesis of 3-(2,4-disubstitutedphenyl)-1-(4-substitutedphenyl)-4-(4,5-diphenyl-1*H-*imidazol-2-yl)-1*H-*pyrazole

Brahmbhatt et al. [2] synthesized 3-(2,4-disubstituted phenyl)-1-(4-substituted phenyl)-4-(4,5-diphenyl-1H-imidazol-2-yl)-1H-pyrazole (Scheme 4). The antibacterial activity of these derivatives was evaluated against *Staphylococcus aureus, Bacillus subtilis*, *Escherichia coli*, and *Pseudomonas aeruginosa* using amikacin sulfate, ampicillin, and chloramphenicol as a reference drug. Compound 4 h shows the most potent activity as compared to the rest of the synthesized compounds. The conclusion of antibacterial activity was presented in (Table 4, Brahmbhatt et al. [2]).

**Table 4:** Antibacterial activity of tri-substituted imidazole derivatives (4a-n) Brahmbhatt et al. [2]

Compounds	Antibacterial activity (MIC in (µg/mL)			
	Gram negative bacteria		Gram positive bacteria	
	*E. coli*	*P. aeruginosa*	*B. subtilis*	*S. aureus*
4a	125.0	125.0	125.0	125.0
4b	125.0	125.0	125.0	125.0
4c	125.0	125.0	125.0	125.0
4d	125.0	125.0	125.0	125.0
4e	125.0	125.0	125.0	125.0
4f	125.0	125.0	125.0	125.0
4g	125.0	125.0	125.0	125.0
4h	125.0	125.0	*31.0*	*63.0*
4i	125.0	125.0	125.0	125.0
4j	125.0	125.0	125.0	125.0
4k	125.0	125.0	125.0	125.0
4l	125.0	125.0	125.0	125.0
4m	125.0	125.0	125.0	125.0
4n	125.0	63.0	125.0	125.0
Amikacin sulphate	2.44	9.77	9.77	9.77
Ampicillin	100	100	–	250
Chloramphenicol	50	50	–	50

Values written in italic signify the best antibacterial activity

**Scheme 5:**
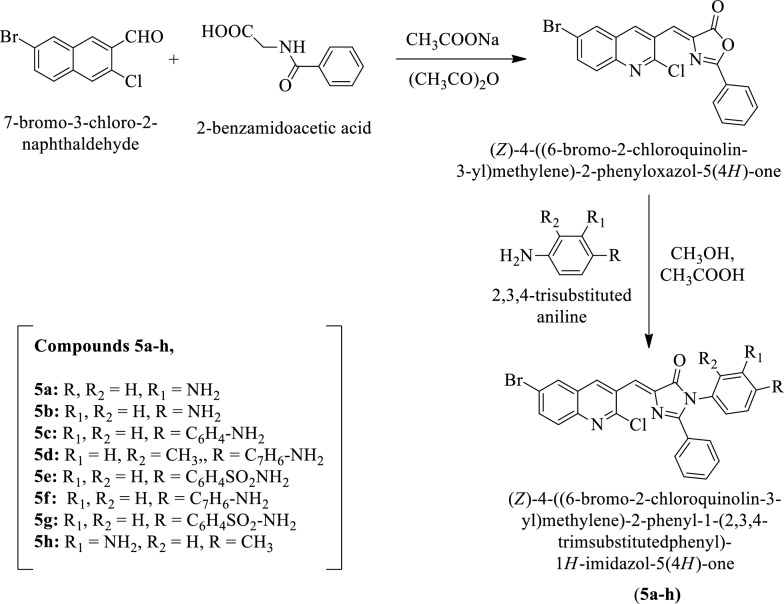
Synthesis of (*Z*)-4-((6-bromo-2-chloroquinonlin-3-yl)methylene)-2-phenyl-1-(2,3,4-trimsubstitutedphenyl)1*H-*imidazol-5(4*H*)-one

Parab et al. [29] synthesized (Z)-4-((6-Bromo-2-chloroquinolin-3-yl) methylene)-2-phenyl-1-(2, 3, 4-trisubstituted phenyl)-1H-imidazol-5(4H)-one by using Scheme 5. The antibacterial activity of synthesized derivatives was evaluated against *E. coli*, *P. aeruginosa, B. subtilis*, and *B. megaterium* by agar cup borer method using streptomycin as a reference drug. The antimycotic potential was evaluated for these derivatives against *Candida albicans* and *Aspergillus niger* using imidil as a reference drug and the conclusion of activity was presented in (Table 5, Parab et al. [29]).

**Table 5:** Antimicrobial activity of synthesized compounds (5a-h) Parab et al. [29]

Compounds	Zone of inhibition (mm)					
	*E. coli*	*P. aeruginosa*	*B. subtilis*	*B. megaterium*	*A. niger*	*C. albicans*
5a	15	19	21	19	20	19
5b	11	9	19	20	10	11
5c	20	22	22	22	13	13
5d	11	15	19	13	15	12
5e	10	8	15	19	12	9
5f	6	18	25	20	14	16
5g	14	9	24	15	10	11
5h	8	13	21	13	16	17
Streptomycin	28	32	31	29	33	33
Imidil	–	–	–	–	34	34

Antimicrobial activity of compounds at 10 mg% in DMSO

**Scheme 6:**
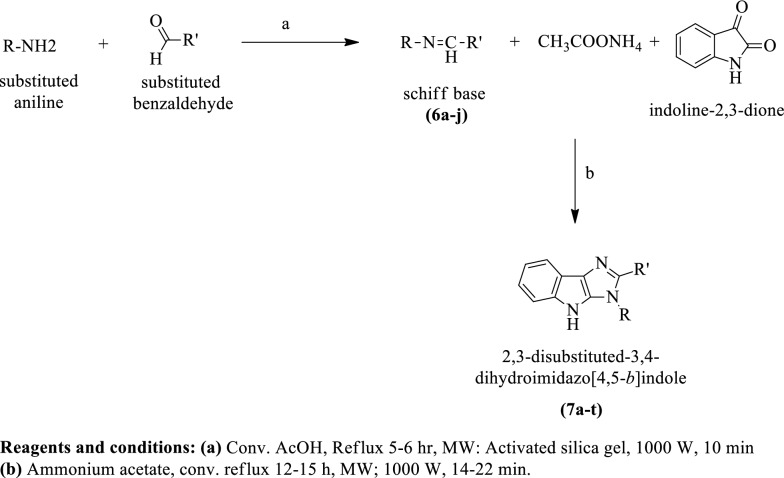
Synthesis of 2,3-disubstituted-3,4-dihydroimidazo[4,5-*b*]indole

Sharma et al. [17] synthesized 2,3-disubstituted-3, 4-dihydroimidazo [4,5-b] indole (Scheme 6) and evaluated for antibacterial activity against *Staphylococcus aureus, Bacillus subtilis*, *Escherichia coli*, and *Klebsiella pneumoniae* by Kirby-Bauer disc technique using ciprofloxacin as reference drug. The conclusion of antimicrobial potential was presented in (Table 6, Sharma et al. [17]).

**Table 6:** Antimicrobial activity of the synthesized aryl imidazole compounds (7a-t) Sharma et al. [17]

Compounds	Diameter of zone of inhibition (mm) Bacterial strains			
	Gram positive bacteria		Gram negative bacteria	
	*S. aureus*	*B. subtilis*	*E. coli*	*K. pneumoniae*
7a	5.9 (50)	6.9 (50)	7.2 (50)	8.1 (50)
7b	5.1 (25)	5.5 (25)	8.1 (50)	8.9 (50)
7c	8.6 (25)	8.4 (25)	9.2 (12.5)	9.5 (12.5)
7d	13.1 (50)	12.5 (25)	11.9 (25)	12.5 (6.2)
7e	9.1 (25)	8.8 (50)	7.6 (100)	7.8 (100)
7f	5.7 (100)	5.9 (100)	6.6 (50)	6.9 (50)
7g	12.5 (50)	12.1 (25)	11.9 (25)	11.6 (25)
7h	11.9 (50)	11.3 (25)	10.9 (100)	10.7 (50)
7i	12.1 (25)	13.8 (50)	14.3 (25)	12.5 (50)
7j	13.1 (25)	12.3 (25)	15.4 (12.5)	11.8 (25)
7k	11.2 (50)	12.4 (25)	13.5 (12.5)	9.1 (50)
7l	6.2 (100)	7.2 (100)	9.2 (50)	7.5 (50)
7m	7.2 (100)	8.7 (50)	10.2 (50)	10.3 (25)
7n	10.3 (25)	12.4 (12.5)	14.5 (6.2)	13.3 (12.5)
7o	12.3 (50)	13.6 (25)	14.6 (25)	14.6 (25)
7p	9.1 (100)	8.3 (100)	9.1 (50)	10.2 (25)
7q	6.1 (100)	7.4 (100)	8.3 (50)	6.9 (50)
7r	7.3 (100)	7.4 (100)	9.5 (50)	9.7 (50)
7s	13.2 (25)	14.5 (12.5)	14.6 (12.5)	11.5 (25)
7t	12.4 (25)	12.7 (25)	13.1 (50)	11.1 (50)
Ciprofloxacin	18 (12.5)	19 (6)	19 (12.5)	17 (6)

Values in brackets are MIC values (µg/mL)

**Scheme 7:**
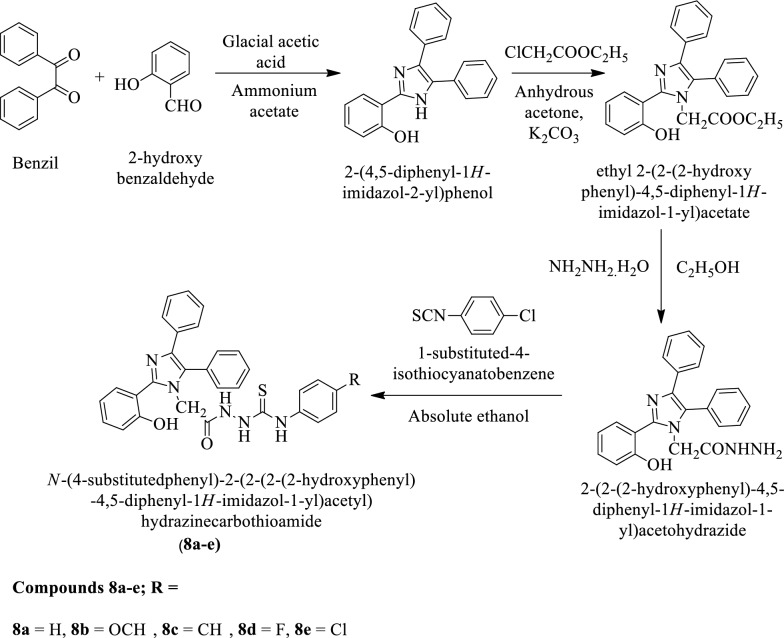
Synthesis of *N*-(4-substitutedphenyl)-2-(2-(2-(2-hydroxyphenyl)-4,5-diphenyl-1*H-*imidazol-1-yl)acetyl)hydrazinecarbothioamide

Ahsan et al. [30] synthesized N-(4-substituted phenyl)-2-(2-(2-(2-hydroxyphenyl)-4, 5-diphenyl-1H-imidazol-1-yl) acetyl) hydrazine carbothioamide (Scheme 7). The antibacterial activity of synthesized derivatives was evaluated against *Escherichia coli*, *Bacillus subtilis*, and *Staphylococcus aureus* using Ofloxacin as a reference drug. The antimycotic potential was evaluated for these derivatives against *C. albicans* using Voriconazole as a positive control. Compounds 8a, 8b, and 8d showed good antifungal activity against *C. albicans.* The conclusion of antimicrobial activity was presented in (Table 7, Ahsan et al. [30]).

**Table 7:** Antibacterial and antifungal activity of titled compounds (8a-8e) Ahsan et al. [30]

Compounds	Antibacterial activity						Antifungal activity	
	*E. coli*		*B. subtilis*		*S. aureus*		*C. albicans*	
	Zone of inhibition (mm)	% inhibition	Zone of inhibition (mm)	% inhibition	Zone of inhibition (mm)	% inhibition	Zone of inhibition (mm)	% inhibition
8a	22	61.11	14	43	14	48	20	75
8b	22	61.11	15	47	21	72	20	69
8c	23	67	15	47	20	69	17	58.6
8d	22.5	62.5	20	62	19	65.5	20	69
8e	19	52	12	50	22	75	20	68.5
Ofloxacin	36	100	32	100	29	100	–	–
Voriconazole	–	–	–	–	–	–	29	100

**Scheme 8:**
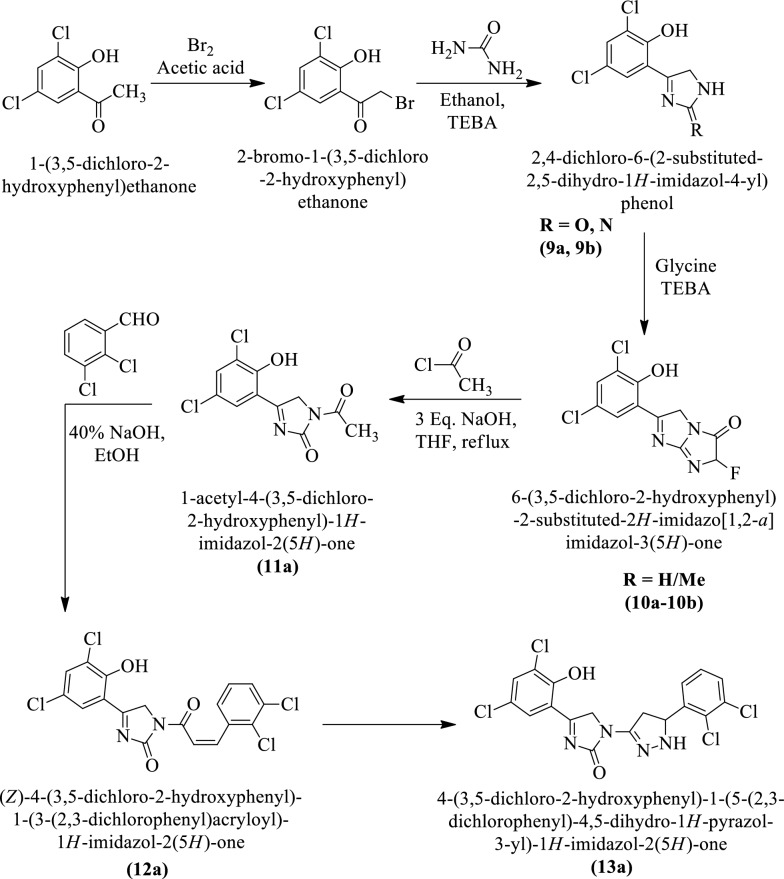
Synthesis of imidazole derivatives

Bhade et al. [18] synthesized 2,4-dichloro-6-(2-substituted-2,5-dihydro-1H-imidazol-4-yl)phenol, 6-(3, 5-dichloro-2-hydroxyphenyl)-2-substituted-2H-imidazo[1,2-a]imidazol-3(5H)-one, 1-acetyl-4-(3, 5-dichloro-2-hydroxyphenyl)-1H-imidazol-2(5H)-one, (Z)-4-(3,5-dichloro-2-hydroxyphenyl)-1-(3-(2, 3-dichlorophenyl) acryloyl)-1H-imidazol-2(5H)-one and 4-(3,5-dichloro-2-hydroxy phenyl)-1-(5-(2,3-dichlorophenyl)-4,5-dihydro-1H-pyrazol-3-yl)-1H-imidazol-2(5H)-one by using (Scheme 8). The antibacterial activity of these derivatives was evaluated against *Staphylococcus aureus, Staphylococcus epidermidis*, *Salmonella typhi* and *Pseudomonas aeruginosa* using chloramphenicol as reference control. The conclusion of activity was presented in (Table 8, Bhade et al. [18]).

**Table 8:** Antibacterial activity of titled compounds (9a-13a) Bhade et al. [18]

Compounds	Gram negative									Gram positive							
	*P. aeruginosa* (MTCC-424)				*S. typhi* (ATCC-25812)					*S. aureus* (ATCC-33591)				*S. epidermidis* (MTCC-3086)			
	AB	SP	ABSP	CL		AB	SP	ABSP	CL	AB	SP	ABSP	CL	AB	SP	ABSP	CL
9a	23	16	26	00		26	19	32	00	16	18	18	00	27	16	28	00
9b	23	16	26	00		27	18	33	00	17	19	17	00	27	15	29	00
10a	23	17	26	00		27	17	33	00	17	20	18	00	27	15	29	00
10b	23	16	25	00		27	18	32	00	17	20	18	00	27	16	28	00
11a	23	12	24	00		27	16	29	00	17	17	18	00	27	15	28	00
12a	22	11	23	00		27	16	30	00	17	16	19	00	27	13	27	00
13a	22	10	23	00		27	15	28	00	16	15	16	00	27	12	28	00

Diameter of inhibition zone (mm) AB-Antibiotic Disc (Chloramphenicol-10), *SP* Sample, *ABSP* Antibiotic + Sample, CL-Control (DMSO), Values were represented as the mean

**Scheme 9:**
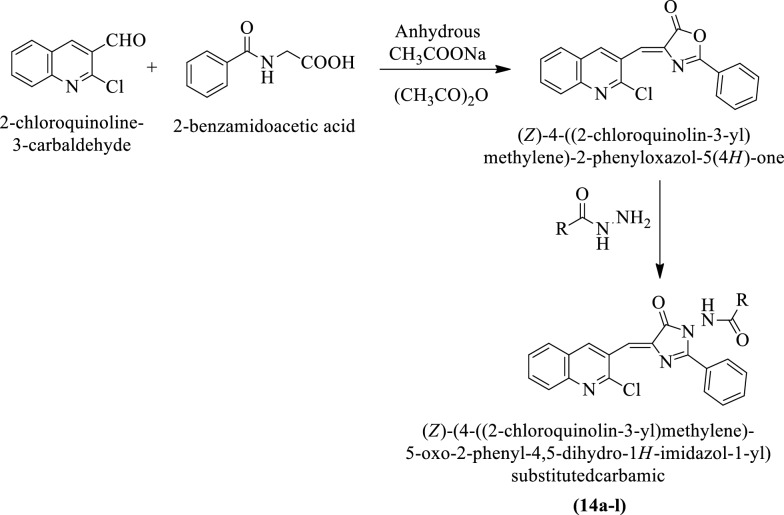
Synthesis of (Z)-(4-((2-chloroquinonlin-3-yl)methylene)-5-oxo-2-phenyl-4,5-dihydro-1*H-*imidazol-1-yl)substitutedcarbamic

Desai et al. [31] synthesized (Z)-(4-((2-chloroquinolin-3-yl)methylene)-5-oxo-2-phenyl-4,5-dihydro-1H-imidazol-1-yl)substituted carbamic (Scheme 9) and evaluated for antimicrobial potential against *Staphylococcus aureus, Escherichia coli, Pseudomonas aeruginosa,* and *Streptococcus pyogenes* by serial broth dilution method using ampicillin as a reference standard and the results were summarized in (Table 9a, Desai et al. [30]). The antimycotic potential of these derivatives was evaluated against *A. niger*, *C. albicans*, and *A. clavatus* using griseofulvin as a reference standard. The results of the activity were summarized in (Table 9b, Desai et al. [31]).

**Table 9:** (a) Antibacterial activity of the synthesized derivatives (14a-l); (b) Antifungal activity of titled compounds (14a-l) Desai et al. [31]

(a)					
Compounds	R	MIC (µg/mL) ± SD			
		*E. coli *MTCC-443	*P. aeruginosa *MTCC-1688	*S. aureus *MTCC-96	*S. pyogenes*MTCC-442
14a	−C_6_H_5_	100 ± 2.03**	500 ± 2.64*	1000 ± 3.78	500 ± 2.64
14b	C_6_H_5_-CH_2_-	500 ± 3.46*	500 ± 3.46	250 ± 3.21**	250 ± 3.04***
14c	−3-Cl-C_6_H_4_	50 ± 2.64***	100 ± 1.21**	200 ± 2.08*	1000 ± 4.51
14d	−4-Cl-C_6_H_4_	25 ± 1*	100 ± 1.51*	200 ± 2.08**	50 ± 2.64**
14e	−2,5-(Cl)_2_-C_6_H_3_	100 ± 1	250 ± 2.51**	1000 ± 4.04	1000 ± 2.51*
14f	−4-F-C_6_H_4_	200 ± 1.62*	100 ± 1.60	100 ± 2.78**	1000 ± 3.78**
14 g	−3-NO_2_-C_6_H_4_	100 ± 1**	100 ± 1.72	500 ± 3.05	250 ± 2.51***
14 h	−4-NO_2_-C_6_H_4_	25 ± 1.62***	50 ± 1.05*	250 ± 2.16*	100 ± 1.78**
14i	−2-OH-C_6_H_4_	100 ± 2.15*	100 ± 1***	100 ± 2.04*	500 ± 4.50
14j	−3-OH-C_6_H_4_	100 ± 2.05*	50 ± 1.16**	500 ± 4.50	200 ± 2.05*
14 k	−2-OH,4-Cl-C_6_H_3_	200 ± 2.21*	100 ± 2.15**	250 ± 2.64**	500 ± 3.08
14 l	C_5_H_4_N	500 ± 3.05**	500 ± 3.78	250 ± 3.21*	100 ± 1.51*
Ampicillin		100 ± 2.05	100 ± 1.0	250 ± 1.52	100 ± 2.06

(b)				
Compounds	R	MIC (µg/mL) ± SD		
		*C. albicans *MTCC-227	*A. niger *MTCC-282	*A. clavatus *MTCC-1323
14a	−C_6_H_5_	500 ± 2.64*	500 ± 3.05*	1000 ± 3.21
14b	C_6_H_5_-CH_2_-	1000 ± 1.04**	1000 ± 2.51**	500 ± 4.05*
14c	−3-Cl-C_6_H_4_	100 ± 1.51*	1000 ± 4.50	100 ± 1.64*
14d	−4-Cl-C_6_H_4_	200 ± 2.64*	100 ± 1.21**	500 ± 4.16
14e	−2,5-(Cl)_2_-C_6_H_3_	100 ± 2.51**	500 ± 2.08***	500 ± 3.78**
14f	−4-F-C_6_H_4_	100 ± 1.78*	1000 ± 3.05	100 ± 2.78***
14 g	−3-NO_2_-C_6_H_4_	200 ± 3.51	500 ± 4.05*	100 ± 1.51*
14 h	−4-NO_2_-C_6_H_4_	100 ± 3.78**	100 ± 1***	200 ± 3.05**
14i	−2-OH-C_6_H_4_	500 ± 4.50*	250 ± 3.78**	500 ± 4.58
14j	−3-OH-C_6_H_4_	1000 ± 2.05***	100 ± 2.05***	500 ± 3.21**
14 k	−2-OH,4-Cl-C_6_H_3_	500 ± 2.08	250 ± 2.05	500 ± 3.46
14 l	C_5_H_4_N	200 ± 3.51**	500 ± 2.64*	100 ± 1.12*
Griseofulvin		500 ± 2.58	100 ± 1	100 ± 1.15

± SD = Standard deviation

^*^Significant *P* < 0.05

^**^Moderately significant *P* < 0.01

^***^Extremely significant *P* < 0.001

**Scheme 10:**
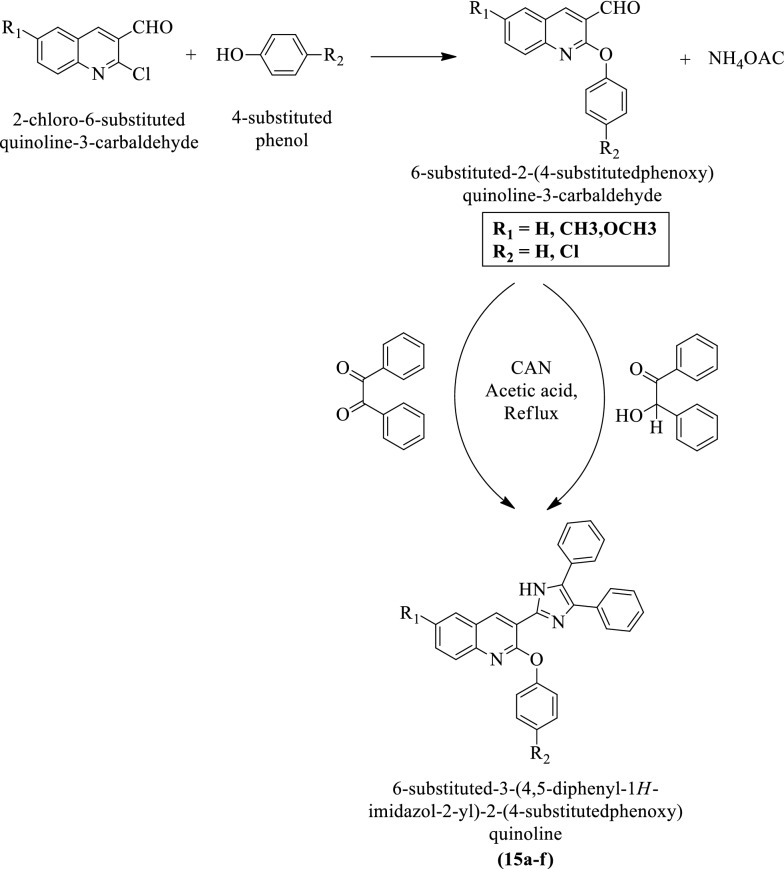
Synthesis of 6-substituted-3-(4,5-diphenyl-1*H-*imidazol-2-yl)-2-(4-substitutedphenoxy)quinoline

Shobhashana et al. [32] synthesized 6-substituted-3-(4,5-diphenyl-1H-imidazol-2-yl)-2-(4-substituted phenoxy) quinoline by using (Scheme 10) and evaluated for antimicrobial activity against *Bacillus subtilis, Escherichia coli*, *Clostridium tetani, Streptococcus pneumoniae*, and *Salmonella typhi* by using the broth dilution method. Ampicillin, chloramphenicol, and ciprofloxacin were used as a positive control. The antimycotic activity of these derivatives was evaluated against *Candida albicans* and *Trichophyton rubrum* using Nystatin and Griseofulvin as reference drugs. The conclusion of antimicrobial activity was presented in (Table 10a, b, Shobhashana et al. [32]).

**Table 10:** (a) Antibacterial activity of the synthesized compounds (15a-f); (b) Antifungal activity of the synthesized compounds (15a-f) Shobhashana et al. [32]

(a)						
Compounds	Minimum inhibitory concentration in µg/mL					
	Antibacterial activity					
	Gram positive bacteria			Gram negative bacteria		
15a	100	250	500	100	250	250
15b	250	500	250	250	200	500
15c	62.5	100	500	62.5	200	250
15d	250	100	125	100	125	100
15e	500	500	500	250	100	500
15f	100	250	100	100	100	250
Ampicillin	250	250	100	100	100	100
Chloramphenicol	50	50	50	50	50	50
Ciprofloxacin	50	100	50	25	25	25

(b)		
Compounds	MIC	
	Antifungal activity	
	*C.albicans *MTCC227	*T. rubrum *MTCC296
15a	> 1000	1000
15b	500	> 1000
15c	1000	1000
15d	1000	1000
15e	1000	> 1000
15f	500	1000
Nystatin	100	500
Griseofulvin	500	500

**Scheme 11:**
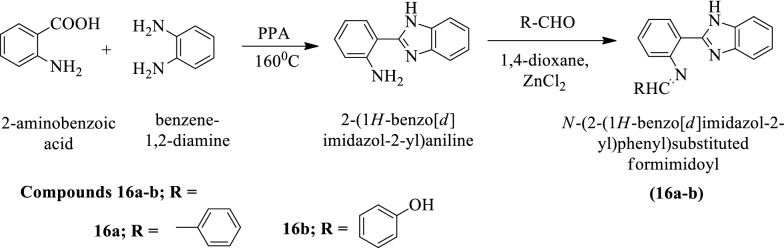
Synthesis of *N*-(2-(1*H*-benzo[*d*]imidazol-2-yl)phenyl)substitutedformimidoyl

Selvan et al. [33] developed N-(2-(1H-benzo[d]imidazol-2-yl)phenyl)substituted formimidoyl by using (Scheme 11). The disc diffusion technique was used for the determination of antimicrobial activity against *S. aureus* using ciprofloxacin as a positive control. The antimycotic activity of these derivatives was evaluated against *A. niger* using Nystatin as a reference drug and the conclusion of antimicrobial potential was presented in (Table 11, Selvan et al. [33]).

**Table 11:** Antimicrobial activity of titled compounds (16a-b) Selvan et al. [33]

Compounds	Zone of inhibition in mm	
	Antibacterial activity	Antifungal activity
	*S. aureus* (NCIM-2079)	*A. niger (*NCIM-105)
16a	22	18
16b	16	20
Solvent	–	–
Ciprofloxacin	35	–
Nystatin	–	35

Standard—Ciprofloxacin 5 mg/disc for bacteria. Nystatin 100 units/disc for fungi; Solvent-DMSO

**Scheme 12:**
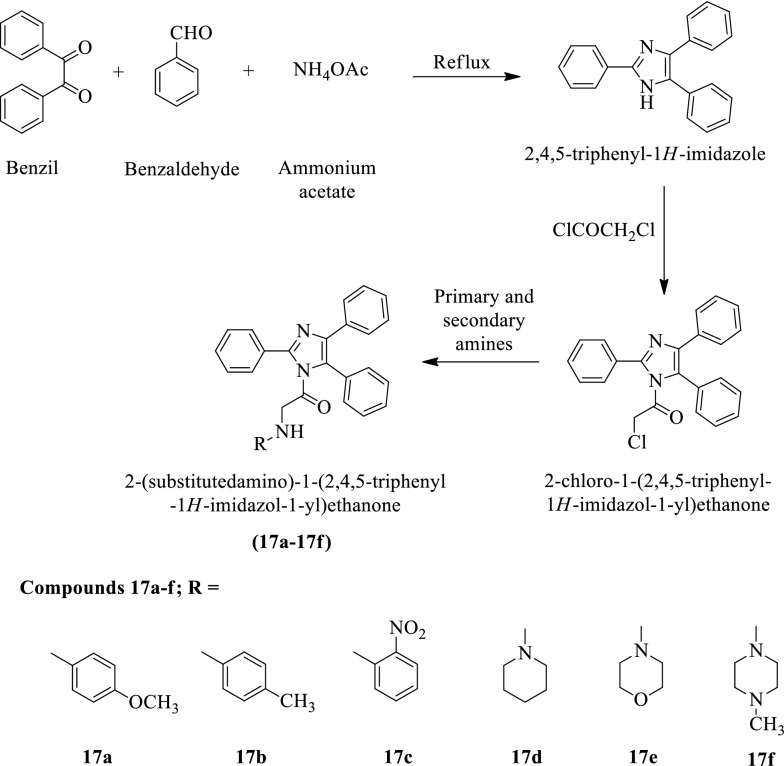
Synthesis of 2-(chloroamino)-1-(2,4,5-tridiphenyl-1*H-*imidazol-1-yl)ethanone

Zala et al. [8] synthesized 2-(substituted amino)-1-(2,4,5-triphenyl-1H-imidazol-1-yl) ethanone (Scheme 12) and evaluated for antimicrobial potential against *Staphylococcus aureus* and *Escherichia coli* using ciprofloxacin as a reference drug. The antimycotic potential of these derivatives was evaluated against *C. albicans* using Clotrimazole as a reference drug. The conclusion of antibacterial activity was presented in (Table 12, Zala et al. [8]).

**Table 12:** Antimicrobial activity of titled compounds (17a-f) Zala et al. [8]

Compounds	Concentration (µg/mL)	Zone of inhibition (mm)		
		Gram positive	Gram negative	Fungi
		*S. aureus*	*E. coli*	*C. albicans*
17a	750	9	10	9
	500	8	9	7
	250	5	6	5
17b	750	16	15	15
	500	12	11	11
	250	10	8	9
17c	750	26	25	21
	500	24	23	19
	250	20	19	18
17d	750	15	16	17
	500	13	14	15
	250	11	10	12
17e	750	17	13	19
	500	14	11	13
	250	12	9	10
17f	750	9	10	15
	500	7	8	13
	250	5	6	10
Ciprofloxacin	750	27	28	–
	500	26	27	–
	250	24	25	–
Clotrimazole	750	–	–	22
	500	–	–	20
	250	–	–	19

**Scheme 13:**
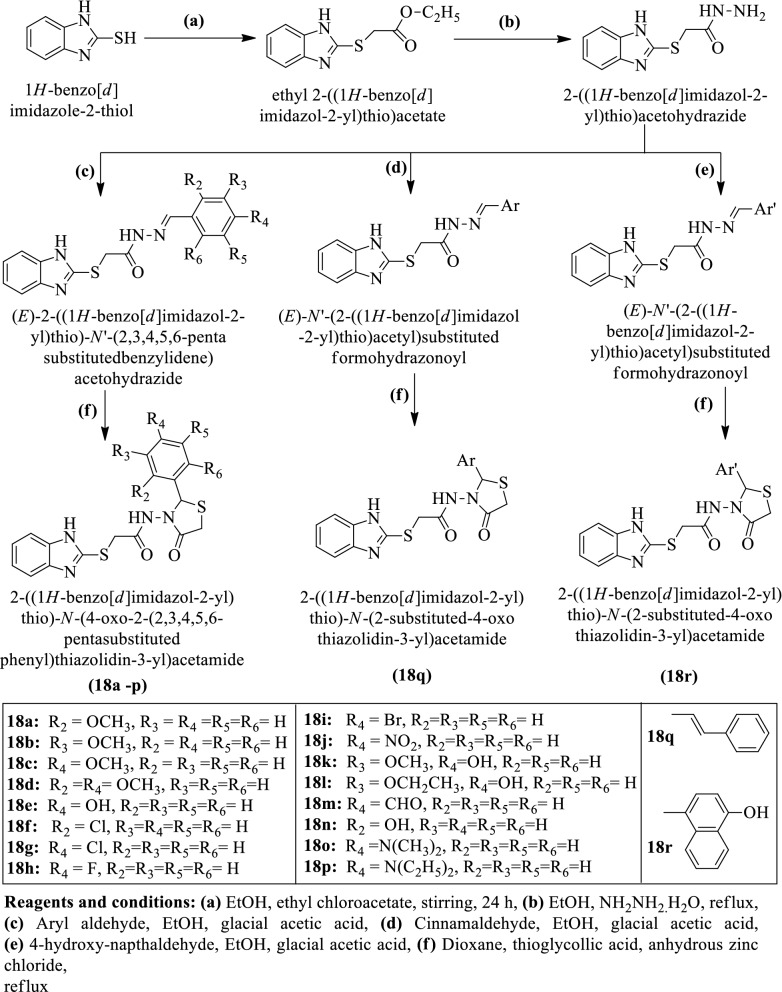
Synthesis of benzimidazole-substituted-1,3-thiazolidin-4-ones

Yadav et al. [34] synthesized 2-((1H-benzo[d]imidazol-2-yl)thio)-N-(4-oxo-2-(2,3,4,5,6-Penta substituted phenyl)thiazolidin-3-yl)acetamide and 2-((1H-benzo[d]imidazol-2-yl)thio)-N-(2-substituted-4-oxothiazolidin-3-yl) acetamide by using (Scheme 13). The antibacterial activity of these derivatives was evaluated against different bacterial strains (*Staphylococcus aureus, Escherichia coli, and Bacillus subtilis*) using Norfloxacin as a reference drug. The antimycotic activity of these derivatives was evaluated against different fungal (*Candida albicans* and *Aspergillus niger)* strains using Fluconazole as a reference drug. The conclusion of the activity was presented in (Table 13, Yadav et al. [34]).

**Table 13:** MIC of benzimidazole-substituted-1,3-thiazolidin4-ones (18a-r) in µM/ml Yadav et al. [34]

Compounds	MIC (µM/ml)				
	*S. aureus*	*B. subtilis*	*E. coli*	*C. albicans*	*A. niger*
18a	0.030	0.030	0.030	0.060	0.030
18b	0.060	0.030	0.030	0.030	0.030
18c	0.030	0.030	0.030	0.030	0.030
18d	0.028	0.014	0.028	0.028	0.028
18e	0.031	0.031	0.031	0.031	0.031
18f	0.030	0.030	0.030	0.030	0.030
18g	0.030	0.015	0.015	0.030	0.030
18h	0.031	0.031	0.031	0.031	0.031
18i	0.027	0.027	0.013	0.027	0.027
18j	0.029	0.029	0.015	0.007	0.029
18k	0.058	0.029	0.007	0.029	0.029
18l	0.028	0.028	0.028	0.028	0.028
18m	0.061	0.030	0.030	0.030	0.030
18n	0.031	0.031	0.008	0.031	0.031
18o	0.029	0.029	0.029	0.029	0.029
18p	0.027	0.027	0.027	0.027	0.027
18q	0.030	0.030	0.030	0.030	0.030
18r	0.028	0.028	0.028	0.028	0.028
Norfloxacin	0.47	0.47	0.47	–	–
Fluconazole	–	–	–	0.50	0.50

### Anticancer activity

**Scheme 14:**
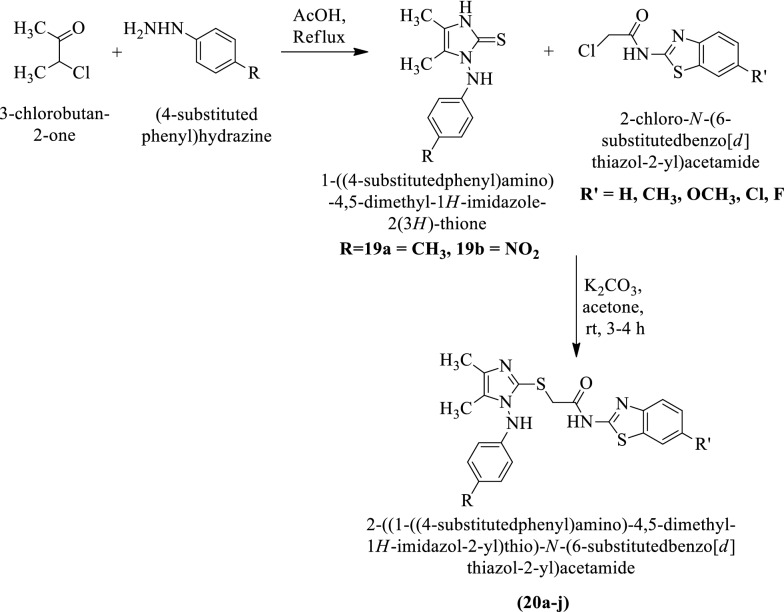
Synthesis of 2-((1-((4-substitutedphenyl)amino)-4,5-dimethyl-1*H-*imidazol-2-yl)thio)-*N*-(6-substitutedbenzo[*d*]thiazol-2-yl)acetamide

Yurttas et al. [35] developed 2-((1-((4-substituted phenyl) amino)-4,5-dimethyl-1H-imidazol-2-yl)thio)-N-(6-substitutedbenzo[d]thiazol-2-yl)acetamide by using (Scheme 14) and evaluated for antitumor potential by **MTT** assay against two different cancer cell lines such as **C6** (rat glioma) and **HepG2** (human liver) using cisplatin as a reference drug. Among the synthesized derivatives compound **20g** shows good cytotoxic potential. The conclusion of antitumor potential was presented in (Table 14, Yurttas et al. [35]).

**Table 14:** IC_50_ values of the synthesized compounds (20a-j) against C6 and HepG2 cancer cell line Yurttas et al. [35]

Compounds	IC_50_ value	
	C6	HepG2
20a	27.0 ± 1.41	50.0 ± 5.0
20b	20 ± 2.0	26.33 ± 1.53
20c	32.67 ± 6.43	275.0 ± 35.36
20d	22.0 ± 3.61	29.33 ± 1.15
20e	16.33 ± 2.31	31.67 ± 7.23
20f	19.50 ± 2.12	28.67 ± 1.15
20g	15.67 ± 2.52	58.33 ± 2.89
20h	> 500	> 500
20i	24.33 ± 4.04	> 500
20j	19.33 ± 2.31	> 500
Cisplatin	23.0 ± 1.73	46.67 ± 7.64

**Scheme 15:**
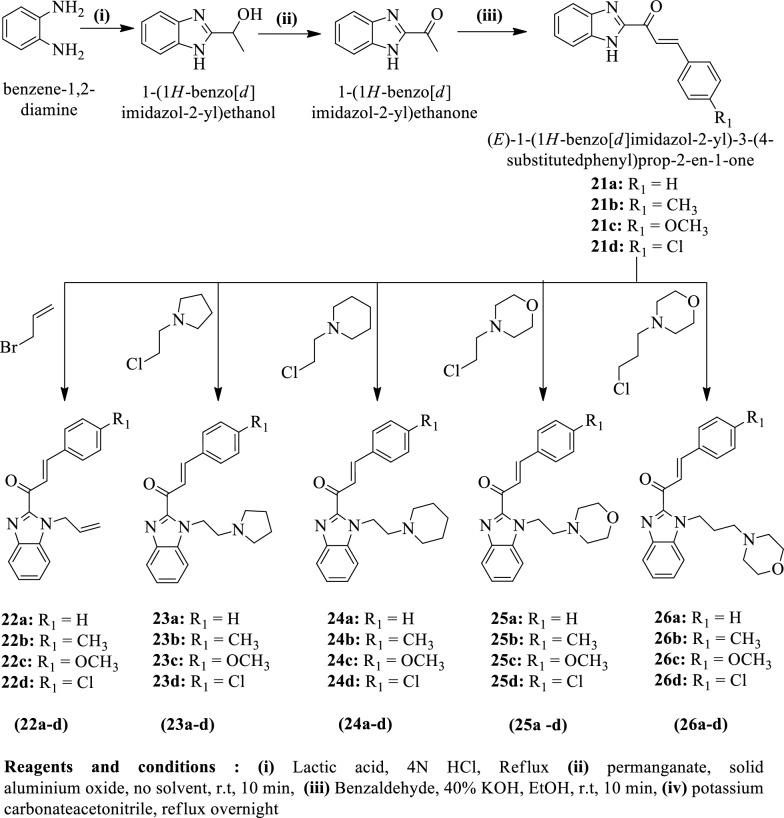
Synthesis of imidazole derivatives

Hsieh et al. [25] synthesized (E)-1-(1-allyl-1H-benzo[d]imidazol-2-yl)-3-(4-substituted phenyl) prop-2-en-1-one by using (Scheme 15) and evaluated for anticancer activity against different cell lines such as **A549, MCF-7, HepG2,** and **OVCAR-3** by **MTT** assay using cisplatin as a reference drug. The conclusion of anticancer activity was presented in (Table 15, Hsieh et al. [25]).

**Table 15:** Anticancer activity of titled compounds (21a-26d) against different cancer cell lines Hsieh et al. [25]

Compounds	Cancer cells (IC_50_ µM)			
	A549	MCF-7	HEP-G2	OVCAR-3
21a	119.3 ± 29.9	13.49 ± 0.16	24.2 ± 0.32	16.91 ± 0.37
21b	19.17 ± 0.43	18.09 ± 0.28	59.13 ± 0.92	24.7 ± 1.69
21c	17.41 ± 0.16	16.04 ± 0.24	140.85 ± 0.88	34.44 ± 1.55
21d	35.89 ± 0.84	32.55 ± 3.26	36.54 ± 1.35	36.48 ± 1.36
22a	12.47 ± 0.18	12.12 ± 0.10	15.44 ± 0.25	16.09 ± 0.39
22b	41.05 ± 1.61	53.54 ± 1.12	117.28 ± 2.42	59.01 ± 8.91
22c	> 314	254.9 ± 13.6	> 314	299.52 ± 9.27
22d	15.79 ± 0.49	13.42 ± 0.24	17.6 ± 0.25	16.13 ± 0.32
23a	10.3 ± 0.13	9.65 ± 0.06	10.16 ± 0.08	10.5 ± 0.10
23b	54.12 ± 1.20	53.19 ± 0.77	64.91 ± 0.24	28.71 ± 1.44
23c	56.21 ± 0.96	56.09 ± 0.14	36.61 ± 1.89	11.4 ± 0.24
23d	19.53 ± 0.71	14.73 ± 0.09	15.49 ± 0.16	14.04 ± 0.29
24a	10.73 ± 0.58	9.73 ± 0.16	10.33 ± 0.06	10.34 ± 0. 19
24b	11.64 ± 0.25	11.14 ± 0.07	32.16 ± 1.83	12.55 ± 0.12
24c	22.36 ± 0.54	21.12 ± 0.53	58.74 ± 0.75	13.29 ± 0.47
24d	50.45 ± 0.82	54.41 ± 0.72	56.45 ± 0.86	33.13 ± 0.14
25a	14.59 ± 0.40	10.38 ± 0.08	36.13 ± 0.75	22.44 ± 0.47
25b	10.76 ± 0.29	10.15 ± 0.06	42.05 ± 0.91	16.32 ± 0.45
25c	10.27 ± 0.15	11.12 ± 0.20	50.24 ± 0.88	14.88 ± 0.67
25d	24.06 ± 0.08	22.93 ± 0.49	21.38 ± 0.68	0.14.22 ± 0.33
26a	9.73 ± 0.07	8.91 ± 0.07	10.93 ± 0.10	10 .76 ± 0.12
26b	11.79 ± 0.27	11.34 ± 0.17	47.88 ± 0.76	13.76 ± 0.27
26c	16.92 ± 0.61	11.93 ± 0.14	32.92 ± 0.38	13.4 ± 0.33
26d	81.48 ± 1.40	35.69 ± 0.47	95.7 ± 2.44	42.24 ± 2.43
DOX	0.46 ± 0.01	0.42 ± 0.01	0.72 ± 0.01	3.95 ± 0.09
Cisplatin	7.31 ± 0.44	11.7 ± 0.12	3.97 ± 0.04	16.04 ± 0.74

**Scheme 16:**
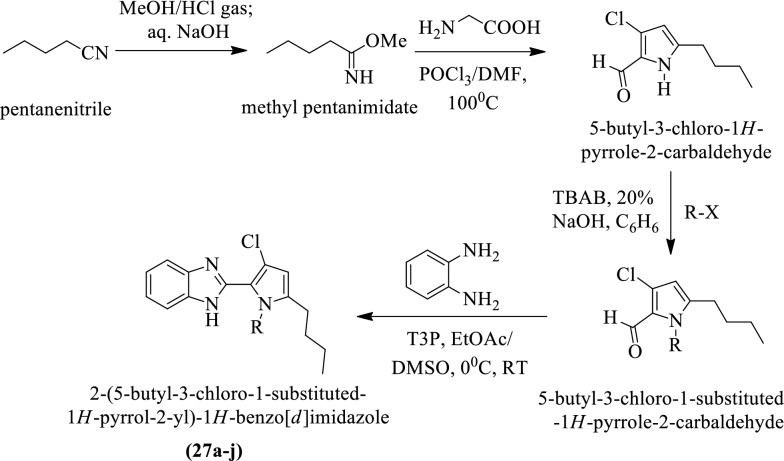
Synthesis of 2-(5-butyl-3-chloro-1-substituted-1*H*-pyrrol-2-yl)-1*H*-benzo[*d*]imidazole

Roopashree et al. [36] synthesized 2-(5-butyl-3-chloro-1-substituted-1H-pyrrol-2-yl)-1H-benzo[d]imidazole (Scheme 16) and evaluated for antitumor activity against **HeLa** cancer cell line by using **MTT** assay. Each compound was tested to calculate the inhibitory concentration and the results of the activity were presented in (Table 16, Roopashree et al. [36]).

**Table 16:** IC_50_ values of the synthesized compounds (27a-j) Roopashree et al. [36]

Compounds	R-X-5	R(6)	IC_50_(µM) ± SD
27a	CH_3_I	CH_3_	> 50
27b	EtBr	Et	> 50
27c	CH_3_(CH_2_)_2_CH_2_Br	CH_3_(CH_2_)_2_CH_2_	> 50
27d	CH_3_(CH_2_)_5_CH_2_Br	CH_3_(CH_2_)_5_CH_2_	25.3 ± 4.18
27e	3-MeC_6_H_4_CH_2_Br	3-MeC_6_H_4_CH_2_	30.2 ± 2.27
27f	3-MeOC_6_H_4_CH_2_Br	3-MeOC_6_H_4_CH_2_	> 50
27g	4-ClC_6_H_4_CH_2_Br	4-ClC_6_H_4_CH_2_	> 50
27h	3,4-Cl_2_C_6_H_3_CH_2_Br	3,4-Cl_2_C_6_H_3_CH_2_	31.9 ± 4.77
27i	4-FC_6_H_4_CH_2_Br	4-FC_6_H_4_CH_2_	30.0 ± 5.12
27j	C_6_H_5_CH_2_Br	C_6_H_5_CH_2_	> 50
Sorafenib			4.1 ± 0.9

*SD* Standard deviation, *IC*_*50*_ Inhibitory concentration 50%

**Scheme 17:**
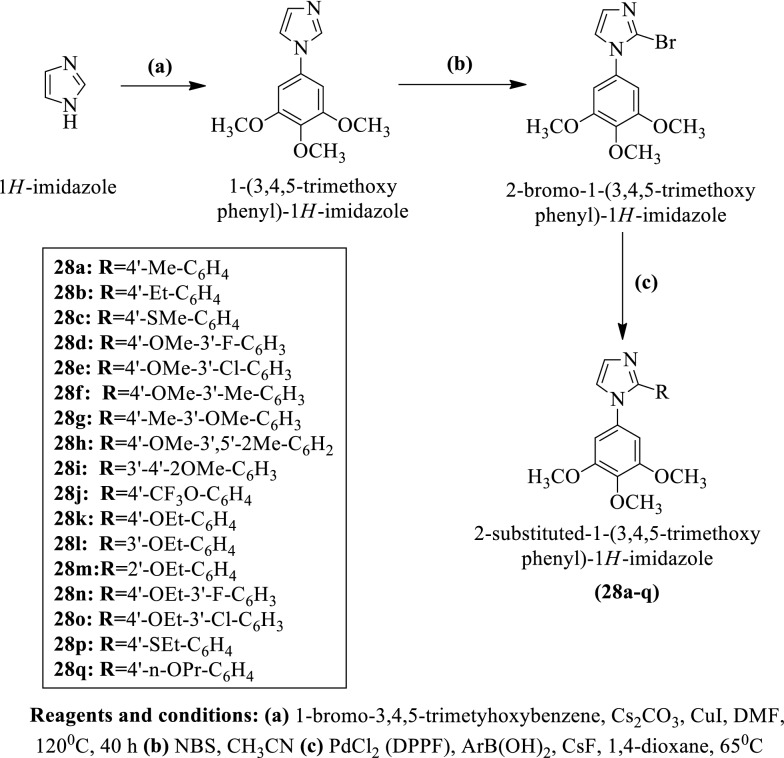
Synthesis of 2-substituted-1-(3,4,5-trimethoxyphenyl)-1*H*-imidazole

Romagnoli et al. [37] developed 2-substituted-1-(3,4,5-trimethoxyphenyl)-1H-imidazole (Scheme 17) and evaluated for anticancer activity against different cancer cell lines such as **HeLa, HT-29, A549, MCF-7, Jurkat,** and **HL-60** using **C-A4** as a reference standard. Compounds **28k, 28n**, and **28o** showed maximum cytotoxicity as compared to others. The conclusion of antitumor potential was presented in (Table 17, Romagnoli et al. [37]).

**Table 17:** Antitumor activity of the synthesized compounds (28a-q) Romangoli et al. [37]

Compounds	IC_50_ (µM)						
	HeLa	HT-29	A549	MCF-7	Jurkat	RS4-11	HL-60
28a	1260 ± 172	1915 ± 354	4733 ± 328	2800 ± 721	760 ± 136	> 10,000	2100 ± 252
28b	1985 ± 126	1400 ± 200	7000 ± 1153	2090 ± 374	7569 ± 758	5678 ± 259	4800 ± 451
28c	337 ± 48	330 ± 36	5600 ± 352	1363 ± 349.8	407 ± 24	800 ± 58	333 ± 41
28d	51 ± 6.5	112 ± 15	121 ± 56	74 ± 17	90 ± 23	217 ± 46	29 ± 9.5
28e	263 ± 39	647 ± 83	2600 ± 422	666 ± 231	365 ± 25	715 ± 148	453 ± 14
28f	330 ± 25	377 ± 83	4717 ± 509	509 ± 25	136 ± 38	475 ± 106	413 ± 27
28 g	623 ± 98	> 10,000	> 10,000	> 10,000	4933 ± 536	2567 ± 784	> 10,000
28 h	9157 ± 1593	> 10,000	> 10,000	> 10,000	> 10,000	> 10,000	3466 ± 467
28i	> 10,000	> 10,000	> 10,000	> 10,000	> 10,000	> 10,000	3933 ± 517
28j	> 10,000	> 10,000	> 10,000	> 10,000	6633 ± 338	> 10,000	> 10,000
28 k	3.7 ± 0.12	1.8 ± 0.8	1.9 ± 1.0	1.5 ± 0.2	1.2 ± 0.5	34.7 ± 0.0	4.8 ± 1.9
28 l	> 10,000	> 10,000	> 10,000	> 10,000	> 10,000	> 10,000	> 10,000
28 m	> 10,000	> 10,000	> 10,000	> 10,000	> 10,000	> 10,000	> 10,000
28n	1.5 ± 0.32	7.5 ± 1.2	14 ± 2.3	3.4 ± 0.38	12 ± 6.6	8.6 ± 1.1	3.5 ± 0.73
28o	3.8 ± 0.7	0.4 ± 0.06	0.57 ± 0.17	0.7 ± 0.06	0.9 ± 0.2	1.2 ± 0.7	1.8 ± 0.6
28p	48 ± 2.5	174 ± 16	228 ± 81	69 ± 7.0	127 ± 27	85 ± 20	12 ± 2.5
28q	2.9 ± 0.8	15 ± 1.3	63 ± 18.1	1.7 ± 0.6	42 ± 3.9	91 ± 8.9	63.0 ± 17.6
CA-4	4 ± 1	180 ± 30	3100 ± 100	5 ± 0.6	0.8 ± 0.2	370 ± 100	1 ± 0.2

**Scheme 18:**
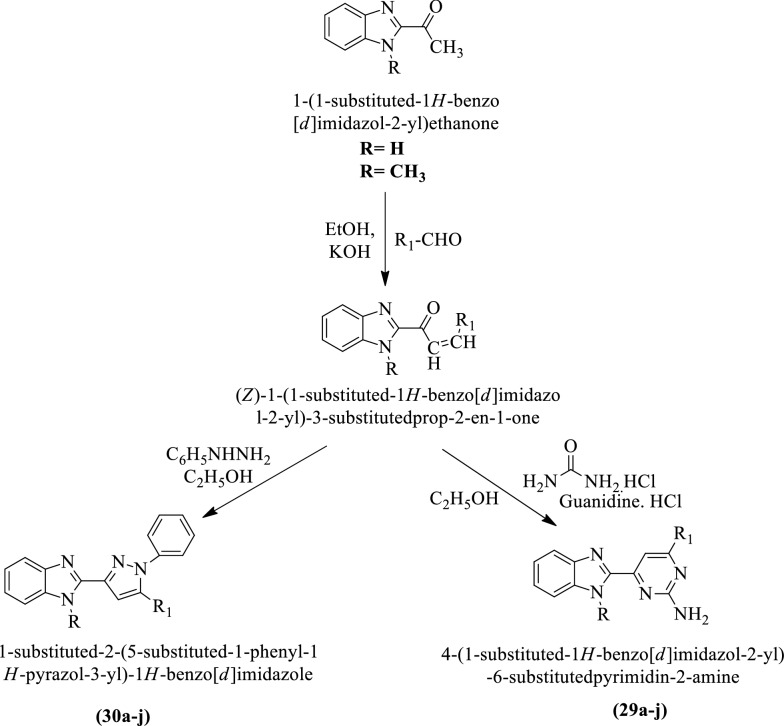
Synthesis of 1-substituted-2-(5-substituted-1-phenyl-1*H*-pyrazol-3-yl)-1*H-*benzo[*d*]imidazole and 4-(1-chloro-1*H-*benzo[*d*]imidazole-2-yl)-6-fluoropyrimidin-2-amine

Rajendran et al. [38] synthesized 1-substituted-2-(5-substituted-1-phenyl-1-H-pyrazol-3-yl)-1H-benzo[d]imidazole and 4-(1-chloro-1H-benzo[d]imidazol-2-yl)-6-fluoropyrimidin-2-amine by using (Scheme 18) and evaluated for antitumor potential against different cell lines such as **MCF-7** and **CaCo-2** using Fluorouracil as reference drug. Each compound was tested to calculate inhibitory concentration and the conclusion of activity was presented in (Table 18a, b, Rajendran et al. [38]).

**Table 18:** (a) IC_50_ of the titled compounds (29a-j) against of MCF-7 and CaCo-2 cell line—benzo [d] imidazole pyrimidine derivatives; (b) IC_50_ of the titled compounds (30a-j) against of MCF-7 and CaCo-2 cell line—benzo [d] imidazole pyrazole derivatives Rajendran et al. [38]

Compounds	Substituent R	Substituent R_1_	Molecular formula	IC50 ± SD (µM)	
				MCF-7	CaCo-2
(a)					
29a	H		C_17_H_13_N_5_	8.22 ± 1.48	5.67 ± 1.25
29b	H		C_16_H_12_N_6_	10.43 ± 1.45	9.56 ± 1.33
29c	H		C_19_H_17_N_5_O_2_	> 30	28.40 ± 2.48
29d	H		C_18_H_14_ClN_5_O	13.05 ± 2.07	12.33 ± 1.80
29e	H		C_25_H_17_N_5_	> 30 ± 2.87	> 30 ± 2.98
29f	CH_3_		C_18_H_15_N_5_	> 30 ± 2.66	> 30 ± 2.43
29 g	CH_3_		C_17_H_14_N_6_	18.56 ± 2.82	16.23 ± 1.24
29 h	CH_3_		C_19_H_16_ClN_5_O	> 30 ± 2.19	25.50 ± 2.74
29i	CH_3_		C_20_H_19_N_5_O_2_	25.11 ± 2.44	21.89 ± 2.35
29j	CH_3_		C_26_H_19_N_5_	> 30 ± 2.80	> 30 ± 2.06
Fluorouracil				7.26 ± 2.30	5.23 ± 2.36

(b)					
30a	H		C_22_H_16_N_4_	22.65 ± 2.32	28.45 ± 2.59
30b	H		C_21_H_15_N_5_	12.79 ± 2.20	9.788 ± 1.48
30c	H		C_23_H_17_ClN_4_O	> 30 ± 2.86	> 30 ± 2.48
30d	H		C_24_H_20_N_4_O_2_	15.34 ± 2.67	13.27 ± 1.56
30e	H		C_30_H_20_N_4_	> 30 ± 2.52	> 30 ± 2.33
30f	CH_3_		C_23_H_18_N_4_	> 30 ± 2.41	> 30 ± 2.69
30g	CH_3_		C_22_H_17_N_5_	19.04 ± 2.56	17.32 ± 2.27
30h	CH_3_		C_24_H_19_ClN_4_O	> 30 ± 2.38	29.76 ± 2.64
30i	CH_3_		C_25_H_22_N_4_O_2_	21.73 ± 2.46	18.35 ± 2.54
30j	CH_3_		C_31_H_22_N_4_	> 30 ± 2.58	> 30 ± 2.62
Fluorouracil				7.26 ± 2.30	5.23 ± 2.36

**Scheme 19:**
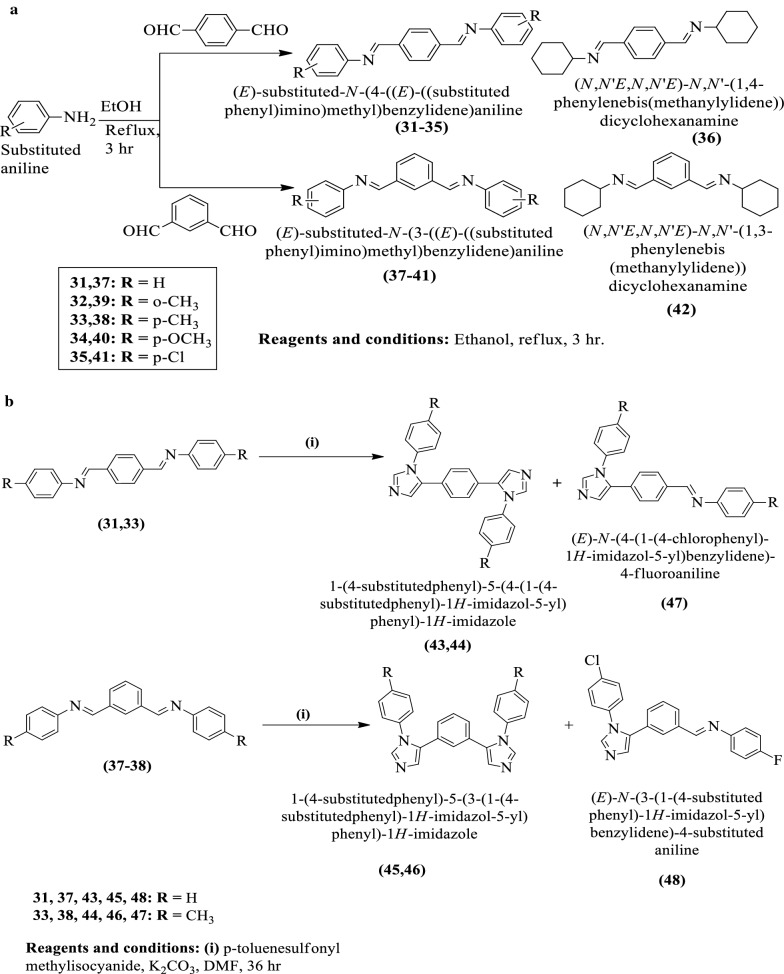

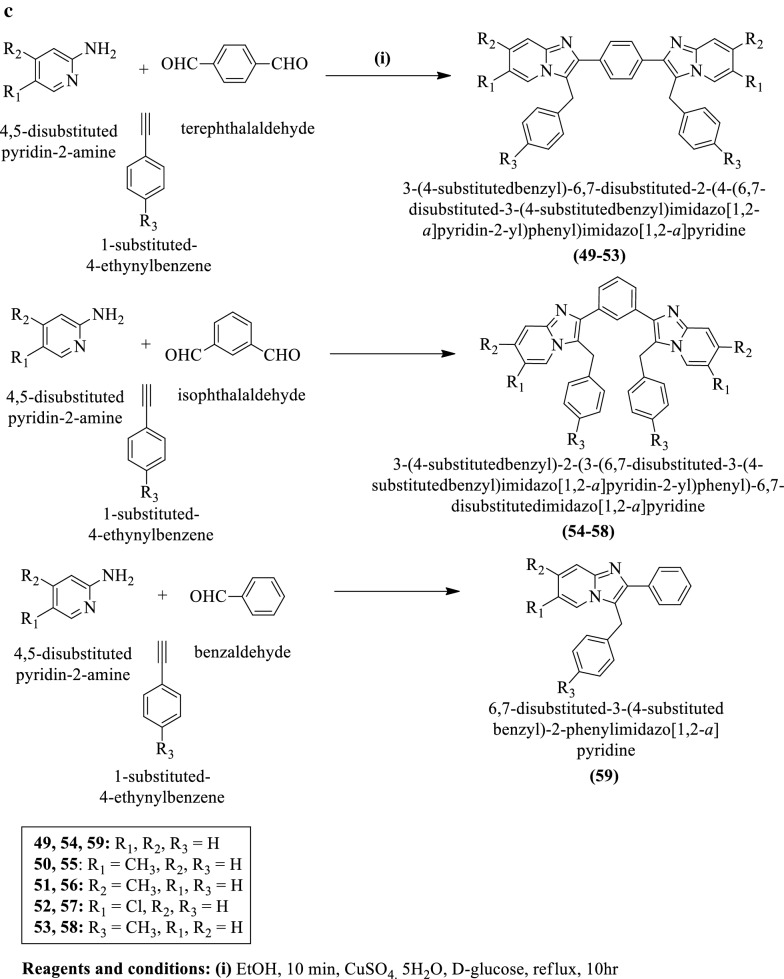
**a** Synthesis of substituted Schiff base; **b** Synthesis of substituted imidazole derivatives; c. Synthesis of substituted phenyl imidazole pyridine derivatives

Meenakshisundaram et al. [39] synthesized 3-(4-substitutedbenzyl)-6,7-disubstituted-2-(4-(6,7-disubstituted-3-(4-substitutedbenzyl) imidazo[1,2-a] pyridin-2-yl)phenyl)imidazo[1,2-a]pyridine, 3-(4-substituted benzyl)-2-(3-(6,7-disubstituted-3-(4-substitutedbenzyl)imidazo[1,2-a]pyridin-2-yl)phenyl)-6,7-disubstitutedimidazo[1,2-a]pyridine and 6,7-disubstituted-3-(4-substitutedbenzyl)-2-phenylimidazo[1,2-a] pyridine (Scheme 19a–c) and evaluated for antitumor potential against different cell lines such as **HeLa, MDA-MB-231** and **ACHN** by **SRB** method using adriamycin as a reference drug. The conclusion of antitumor potential was presented in (Table 19, Meenakshisundaram et al. [39]).

**Table 19:** Anticancer activity of the synthesized derivatives (31–59) against three different cancer cell lines Meenakshisundaram et al. [39]

Compounds	HeLa			MDA-MB-231				ACHN		
	IC_50_ (μM)	TGI (μM)	GI_50_ (μM)	IC_50_ (μM)		TGI (μM)	GI_50_ (μM)	IC_50_ (μM)	TGI (μM)	GI_50_ (μM)
31	> 10	> 10	> 10	> 10		> 10	> 10	> 10	> 10	> 10
32	> 10	> 10	> 10	> 10		> 10	> 10	> 10	> 10	> 10
33	> 10	9.47	> 10	> 10		> 10	> 10	> 10	> 10	> 10
34	> 10	> 10	> 10	> 10		> 10	> 10	> 10	> 10	> 10
35	> 10	> 10	> 10	> 10		> 10	> 10	> 10	> 10	> 10
36	> 10	> 10	> 10	> 10		> 10	> 10	> 10	> 10	> 10
37	> 10	9.79	8.23	> 10		> 10	> 10	> 10	> 10	> 10
38	> 10	9.67	> 10	> 10		> 10	8.90	> 10	> 10	> 10
39	> 10	> 10	9.12	> 10		8.45	> 10	> 10	> 10	7.50
40	> 10	> 10	8.23	> 10		> 10	> 10	> 10	> 10	> 10
41	> 10	> 10	> 10	> 10		> 10	> 10	> 10	> 10	> 10
42	> 10	> 10	> 10	> 10		> 10	> 10	> 10	> 10	> 10
43	> 10	9.76	4.23	> 10		> 10	5.14	> 10	> 10	8.24
44	> 10	9.76	1.86	> 10		> 10	1.16	> 10	> 10	3.78
45	> 10	> 10	> 10	> 10		> 10	6.88	> 10	> 10	9.88
46	> 10	9.76	6.85	> 10		> 10	4.26	> 10	> 10	7.15
47	> 10	> 10	1.92	> 10		> 10	1.20	> 10	> 10	2.24
48	> 10	> 10	3.10	> 10		> 10	1.90	> 10	> 10	3.86
49	> 10	> 10	0.55	> 10		> 10	0.43	> 10	> 10	0.55
50	> 10	> 10	1.20	> 10		> 10	0.88	> 10	> 10	1.16
51	> 10	> 10	2.25	> 10		> 10	2.05	> 10	> 10	1.90
52	> 10	3.73	5.24	> 10		> 10	4.50	> 10	> 10	7.72
53	> 10	9.76	0.96	> 10		> 10	1.30	> 10	> 10	1.32
54	> *10*	> *10*	*0.36*	> *10*		> *10*	*0.30*	> *10*	> *10*	*0.38*
55	> 10	> 10	0.84	> 10		> 10	0.65	> 10	> 10	0.98
56	> 10	> 10	0.97	> 10		> 10	0.58	> 10	> 10	0.85
57	> 10	9.74	4.00	> 10		> 10	1.60	> 10	> 10	1.82
58	> 10	9.76	0.73	> 10		> 10	1.59	> 10	> 10	0.62
59	> 10	> 10	> 10	> 10		> 10	> 10	> 10	> 10	8.24
Adriamycin	> 10	> 10	0.52	> 10		> 10	0.51	> 10	> 10	0.58

*GI*_*50*_ Concentration of drug causing 50%inhibition of cell growth

*IC*_*50*_ Concentration of drug causing 50% cell kill

*TGI* Concentration of drug causing total inhibition of cell growth

Italic values indicate the activity best compounds

Inhibitory activity was expressed in micromolar

**Scheme 20:**
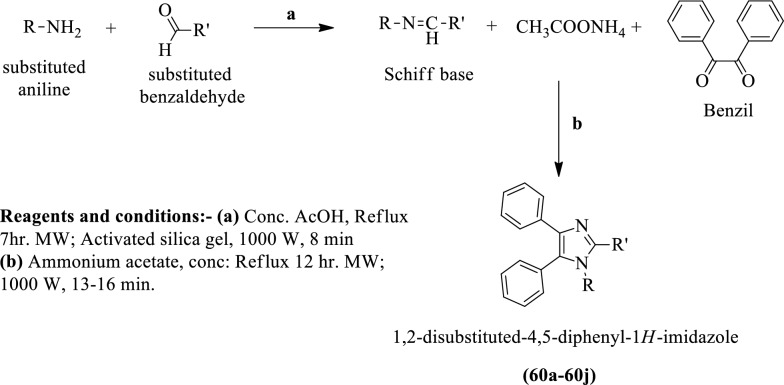
Synthesis of 1,2-disubstituted-4,5-diphenyl-1*H*-imidazole

Sharma et al. [40] synthesized 1,2-disubstituted-4, 5-diphenyl-1H-imidazole (Scheme 20), and evaluated for antitumor potential by using the tryphan blue dye exclusion technique against different cancer cell lines such as **DLA** and **EAC** at different concentration. The conclusion of antitumor potential was presented in (Table 20, Sharma et al. [40]).

**Table 20:** Antitumor activity of the synthesized derivatives (60a-j) Sharma et al. [40]

Compounds	Substituent R	Substituent R’	DLA cells CTC_50_ μg/mL	EAC cells CTC_50_ μg/mL
60a			190.26	60.50
60b			114.00	240.00
60c			98.56	31.25
60d			309.67	200.22
60e			> 500	489.34
60f			207.60	115.31
60			238.50	31.25
60 h			> 500	> 500
60i			405.68	305.91
60j			150.26	94.63

*CTCs* The cytotoxic concentration (which inhibited 50% of total cells)

### Antioxidant activity

**Scheme 21:**
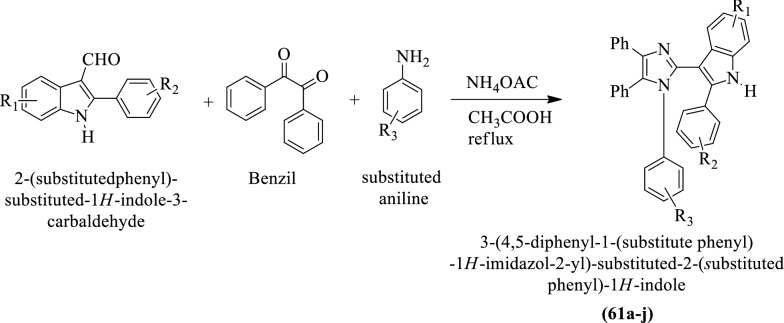
Synthesis of 3-(4,5-diphenyl-1-(substitute phenyl)-1*H*-imidazol-2-yl)-sunstituted-2-(substitutedphenyl)- 1*H*-indole

Naureen et al. [41] synthesized 3-(4,5-diphenyl-1-(substituted phenyl)-1H-imidazol-2-yl)-substituted-2-(substituted phenyl)-1H-indole (Scheme 21) and evaluated for antioxidant potential by **DPPH** method using Quercetin as reference drug. Compound **61d** shows the highest antioxidant activity as compared to others. The conclusion of antioxidant potential was presented in (Table 21, Naureen et al. [41]).

**Table 21:** Antioxidant activity of the synthesized derivatives (61a-j) Naureen et al. [41]

Compounds	R_1_	R_2_	R_3_	Antioxidant activity	
				Inhibition (%) at 0.5 mM	IC_50_ (µM)
61a	H	Cl	CH_3_	62.58 ± 0.7	175.26 ± 1.24
61b	H	Cl	Br	71.74 ± 0.2	146.27 ± 1.09
61c	Br	H	F	71.87 ± 0.5	181.26 ± 1.1
61d	H	Br	CH_3_	90.39 ± 0.5	148.26 ± 1.2
61e	H	Br	Cl	20.97 ± 0.5	–
61f	H	CH_3_	H	67.61 ± 0.3	162.27 ± 1.2
61g	H	CH_3_	CH_3_	44.21 ± 0.7	–
61h	H	CH_3_	Br	7.11 ± 0. 2	–
61i	H	CH_3_	F	18.91 ± 0. 6	–
61j	H	CH_3_	OCH_3_	23.03 ± 0.5	–
Thiourea				–	–
Quercetin				93.21 ± 0.9	16.96 ± 0.1

**Scheme 22:**
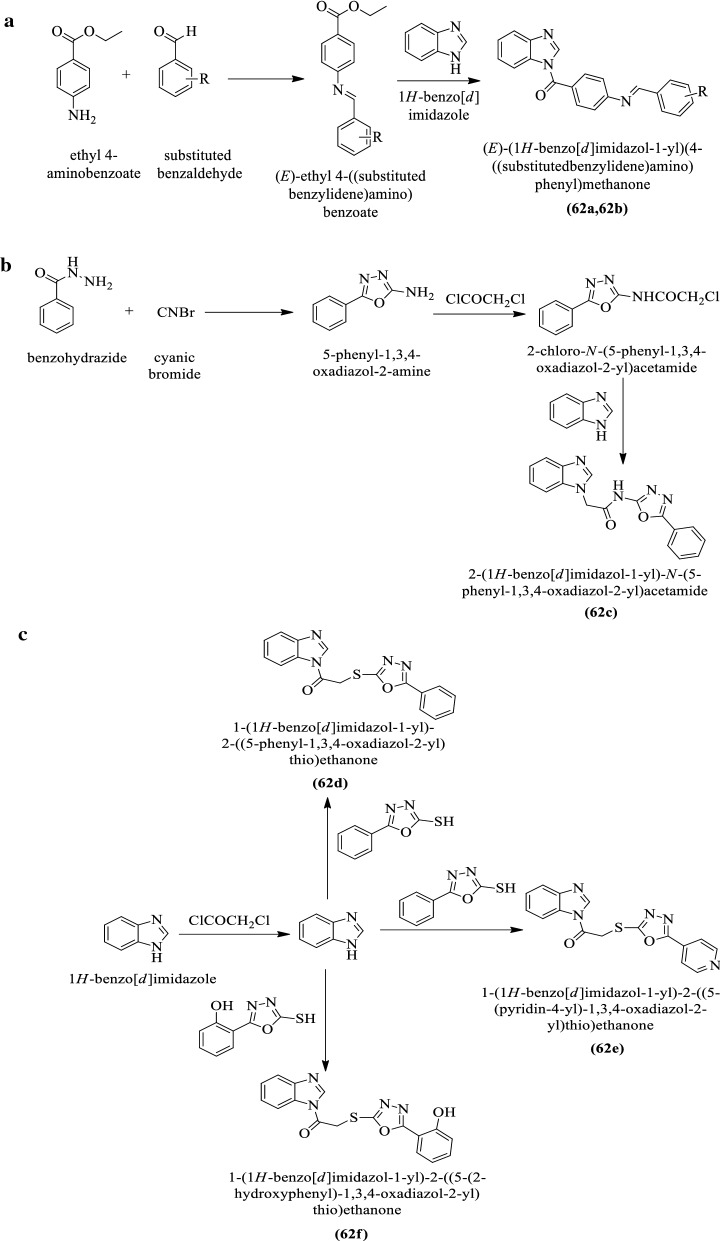
**a** Synthesis of (*E*)-(1*H-*benzol[*d*]imidazole-1-yl)(4-(substitutedbenzylidene)amino)phenyl)methanone. **b** Synthesis of 2-(1*H-*benzol[*d*]imidazole-1-yl)-*N*-(5-phenyl-1,3,4-oxadiazol-2-yl)acetamide. **c** Synthesis of substituted imidazole linked 1,3,4-oxadiazole derivatives

Rajasekaran et al. [42] synthesized (E)-(1H-benzo[d]imidazol-1-yl)(4-((substituted benzylidene)amino)phenyl)methanone (Scheme 22a), 2-(1H-benzo[d]imidazol-1-yl)-N-(5-phenyl-1,3,4-oxadiazol-2-yl)acetamide (Scheme 22b) and 1-(1H-benzo[d]imidazol-1-yl)-2-((substituted-1,3,4-oxadiazol-2-yl)thio)ethanone (Scheme 22c) and evaluated for antioxidant potential by using **DPPH** assay. All the synthesized derivatives showed good scavenging potential as compared to ascorbic acid (positive control) and the conclusion of activity was presented in (Table 22, Rajasekaran et al. [42]).

**Table 22:** Antioxidant activity of the synthesized compounds (62a-f) Rajasekaran et al. [42]

Compounds	% Inhibition			
	10 μg/ml	20 μg/ml	30 μg/ml	40 μg/ml
62a	7.20	12.30	37.65	39.42
62b	34.77	34.66	37.65	39.42
62c	7.08	15.61	21.04	22.26
62d	17.71	29.34	30.34	40.86
62e	34.77	37.76	47.17	52.16
62f	18.98	24.67	28.90	34.34
Ascorbic acid	56.03	58.80	65.33	68.55

**Scheme 23:**
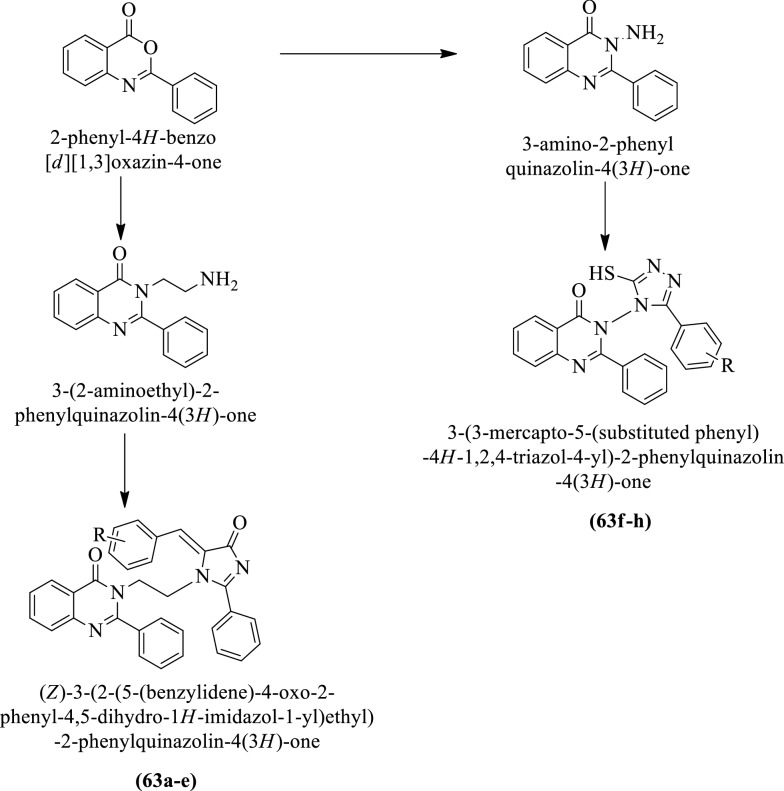
Synthesis of (*Z*)-3-(2-(5-(3-methyl benzylidene)-4-oxo-2-phenyl-4, 5-dihydro-1*H*-imidazol-1-yl) ethyl)-2-phenyl quinazolin-4(3H)-one and 3-(3-mercapto-5-(susbstituted phenyl)-*4H*-1,2,4-triazol-4-yl)-2-phenylquinazolin-4(3*H)*-one

Subramaniam et al. [43] synthesized (Z)-3-(2-(5-(3-methyl benzylidene)-4-oxo-2-phenyl-4, 5-dihydro-1H-imidazol-1-yl) ethyl)-2-phenyl quinazolin-4(3H)-one derivatives (Scheme 23) and evaluated for antioxidant potential by using **DPPH** assay. These compounds showed good scavenging potential as compared to ascorbic acid (positive control). The conclusion of scavenging potential was presented in (Table 23, Subramaniam et al. [43]).

**Table 23:** Antioxidant activity of the synthesized derivatives (63a-h) Subramaniam et al. [43]

Compounds	Concentration (μg/ml)										
	10	20	30	40	50	60	70	80		90	100
63a	2.54	8.47	14.61	20.97	27.86	33.36	42.37	45.12		51.58	56.25
63b	2.11	10.06	19.17	29.34	33.15	40.57	48.62	52.43		62.5	69.70
63c	1.80	10.48	17.05	25.42	33.30	40.57	48.19	55.82		65.36	71.61
63d	1.37	7.41	15.14	20.65	27.33	33.89	39.72	47.35		51.37	59.42
63e	1.80	6.88	14.83	21.29	27.22	33.47	40.25	47.98		51.48	57.83
63f	7.94	21.5	34.32	46.29	59.11	71.61	84.53	97.35		97.98	98.83
63 g	12.71	27.54	40.99	55.40	73.83	84.21	93.53	94.91		95.65	96.71
63 h	10.91	22.77	37.07	51.16	65.14	68.32	89.72	92.37		92.69	95.85
Standard	Concentration (μg/ml)										
	01	02	03	04	05	06	07	08		09	10
Ascorbic acid	8.76	15.34	26.08	37.65	41.23	59.29	67.43	76.53		80.21	87.76

**Scheme 24:**
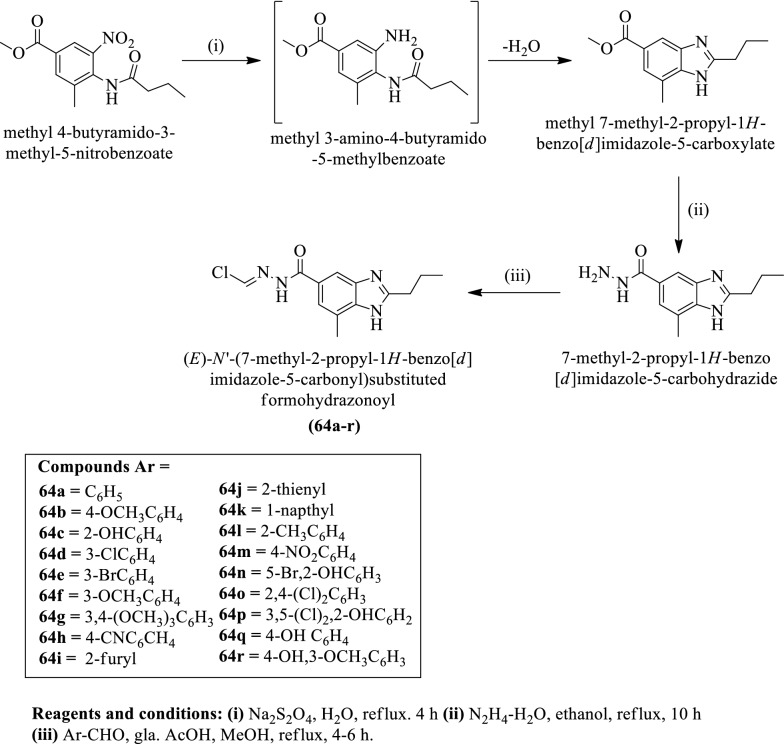
Synthesis of (*E*)-N'-(7-methyl-2-propyl-1H-benzo[*d*]imidazole-5-carbonyl)substituted formohydrazonuyl

Katikireddy et al. [21] developed (E)-N'-(7-methyl-2-propyl-1H-benzo[d]imidazole-5-carbonyl) substituted formohydrazonoyl (Scheme 24) and evaluated for antioxidant activity using ascorbic acid as a reference drug. Compound **64n** shows the most potent antioxidant activity as compared to others and the results of activity were presented in (Table 24, Katikireddy et al. [21]).

**Table 24:** Antioxidant activity of synthesized derivatives (64a-r) Katikireddy et al. [21]

Compounds	IC_50_ (μg/ml)
64a	49.28 ± 3.03
64b	32.17 ± 2.87
64c	29.10 ± 1.60
64d	18.31 ± 1.38
64e	26.81 ± 2.10
64f	29.96 ± 2.81
64g	24.79 ± 3.03
64h	30.83 ± 2.93
64i	23.19 ± 1.72
64j	30.08 ± 2.60
64k	20.05 ± 1.27
64l	25.97 ± 2.18
64m	13.60 ± 1.37
64n	9.40 ± 1.04
64o	12.39 ± 1.26
64p	16.27 ± 1.39
64q	24.70 ± 2.29
64r	38.28 ± 3.07
Ascorbic acid	7.50 ± 0.89

**Scheme 25:**
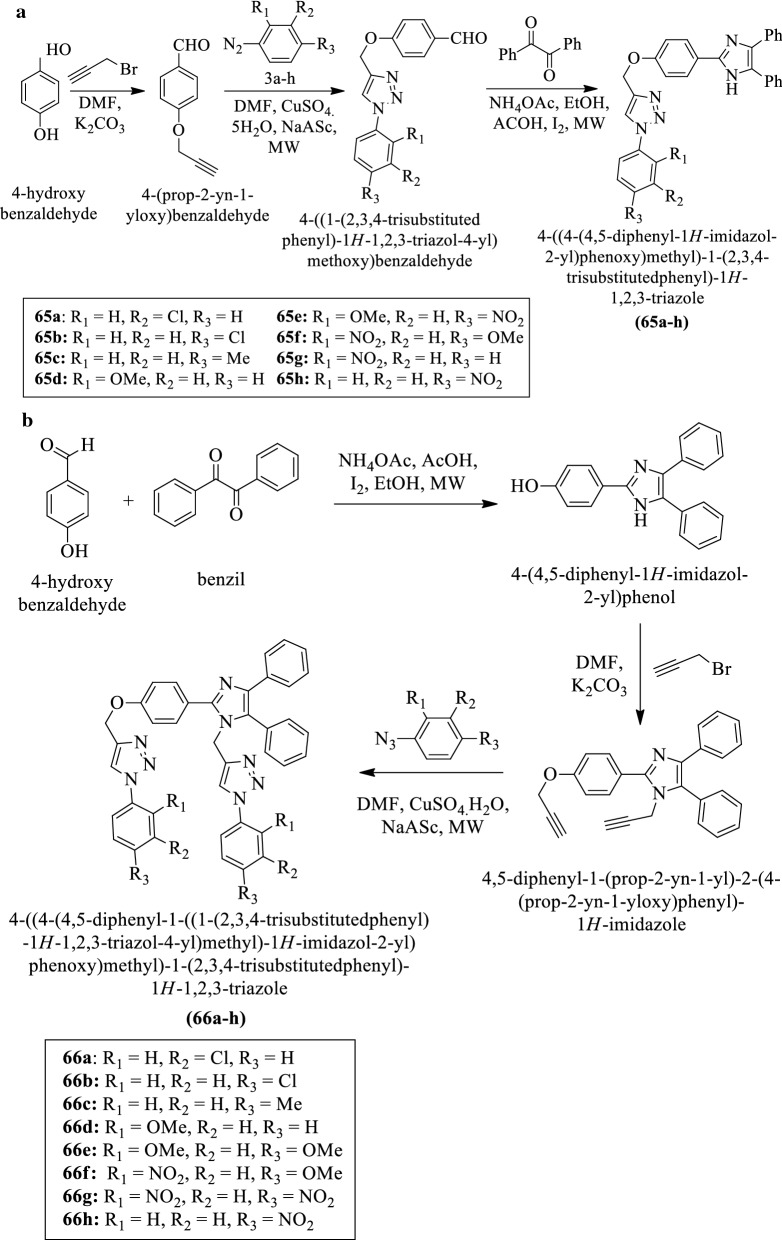
**a** Synthesis of 4-((4-(4,5-diphenyl-1H-imidazol-2-yl)phenoxy)methyl)-1-(2, 3, 4-trisubstituted phenyl)-1H-1, 2, 3-triazole; **b** Synthesis of 4-((4-(4,5-diphenyl-1-((1-(2, 3, 4-trisubstituted phenyl)-1*H*-1, 2, 3-triazole-4-yl)methyl)-1H-imiidazol-2-yl)phenoxy)methyl)-1-(2,3,4-trisubstitutedphenyl)-1*H*-1,2,3-triazole

Subhashini et al. [44] synthesized 4-((4-(4,5-diphenyl-1H-imidazol-2-yl)phenoxy)methyl)-1-(2,3,4-trisubstituted phenyl)-1H-1,2,3-triazole derivatives (Scheme 25a, b) and evaluated for antioxidant activity by using four different methods such as **Hydrogen peroxide scavenging, Nitric oxide scavenging, DPPH,** and **FRAP assay**. The conclusion of antioxidant potential was presented in (Table 25a–d, Subhashini et al. [44]).

**Table 25:** (a) DPPH radical scavenging activity of (65a-h) and (66a-h); (b) Hydrogen peroxide radical scavenging activity of (65a-h) and (66a-h); (c) Nitric oxide radical scavenging activity of (65a-h) and (66a-h); (d) FRAP oxide radical scavenging activity of (65a-h) and (66a-h) Subhashini et al. [44]

Compounds	Concentration			
	10 μg/ml	50 μg/ml	100 μg/ml	250 μg/ml
(a)				
65a	57	71	81	94
65b	49	55	59	63
65c	42	53	65	69
65d	53	59	64	93
65e	35	42	55	63
65f	44	61	79	90
65 g	41	49	53	61
65 h	67	75	83	91
66a	55	63	71	87
66b	60	69	76	89
66c	69	78	81	95
66d	48	67	79	85
66e	71	79	85	96
66f	33	44	55	61
66 g	41	47	59	62
66 h	66	74	81	90
Standard	85	89	93	97

(b)				
65a	49	67	75	87
65b	59	73	81	92
65c	40	49	55	57
65d	47	65	72	89
65e	31	43	49	56
65f	52	73	81	92
65 g	35	43	51	63
65 h	57	68	75	88
66a	51	63	78	91
66b	54	71	82	90
66c	71	88	91	96
66d	57	73	85	94
66e	37	45	52	59
66f	65	78	86	94
66 g	38	45	53	55
66 h	57	65	78	86
Standard	83	91	95	98

(c)				
65a	49	55	63	78
65b	54	69	75	89
65c	31	37	44	51
65d	56	68	79	85
65e	29	36	41	47
65f	48	56	67	74
65 g	23	32	39	43
65 h	61	77	86	95
66a	65	75	82	89
66b	57	69	79	87
66c	68	79	88	91
66d	57	68	75	88
66e	25	37	42	46
66f	48	55	67	78
66 g	21	27	33	39
66 h	67	65	77	86
Standard	81	86	91	96

(d)				
65a	47	64	78	87
65b	51	67	79	93
65c	31	39	43	47
65d	63	77	83	92
65e	22	27	32	38
65f	57	68	77	85
65 g	27	33	40	45
65 h	49	58	69	87
66a	56	63	75	89
66b	49	58	67	85
66c	65	71	84	97
66d	64	79	86	91
66e	30	37	45	50
66f	45	53	62	85
66 g	31	39	42	48
66 h	60	69	78	87
Standard	88	92	95	99

### Antihypertensive activity

**Scheme 26:**
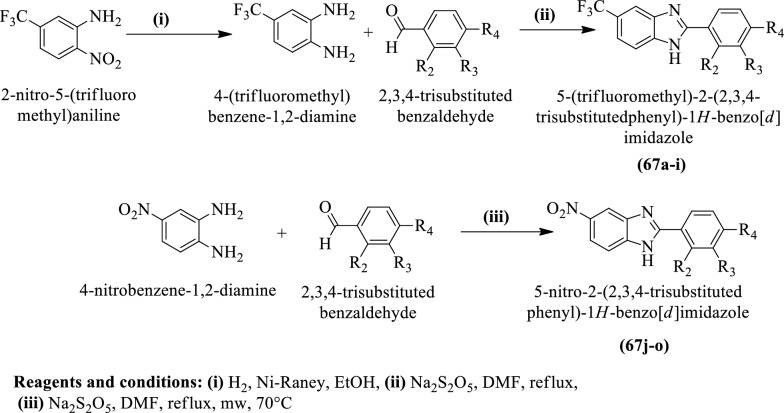
Synthesis of 5-(trifluoromethyl)-2-(2, 3, 4-trisubstituted phenyl)-1*H*-benzo[*d*] imidazole and 5-nitro-2-(2, 3, 4-trisubstituted phenyl)-1*H*-benzo[*d*]imidazole

Navarrete-Vazquez et al. [45] synthesized 5-(trifluoromethyl)-2-(2,3,4-trisubstituted phenyl)-1H-benzo[d] imidazole and 5-nitro-2-(2,3,4-trisubstituted phenyl)-1H-benzo [d] Imidazole (Scheme 26) and evaluated for antihypertensive potential in **SHR** by using the tail-cuff method and the results of antihypertensive activity were summarized in (Table 26, Navarrete-Vazquez et al. [45]).

**Table 26:** Antihypertensive activity of the synthesized derivatives (67a-o) in SHR Navarrete-Vázquez et al. [45]

Compounds	R1	R2	R3	R4	Ex vivo vasorelaxant effect			
					With endothelium (+ E)		Without endothelium (−E)	
					EC50 (μM)	E_max_ (%)	EC50 (μM)	E_max_ (%)
67a	−CF_3_	−H	−H	−H	369.37 ± 10.2	91.2 ± 1.18	467.75 ± 73.6	75.6 ± 6.31
67b	−CF_3_	−OMe	−H	−H	210.33 ± 11.3	75.14 ± 33.5	574.85 ± 30.3	45.7 ± 15.4
67c	−CF_3_	−OEt	−H	−H	548.5 ± 27.8	90.97 ± 2.30	548.51 ± 77.1	19.8 ± 8.13
67d	−CF_3_	−NO_2_	−H	−H	3.18 ± 0.30	93.16 ± 3.52	15.03 ± 7.59	85.31 ± 2.63
67e	−CF_3_	−H	−H	−OH	219.20 ± 14.1	51.15 ± 20.6	219.20 ± 71.6	37.04 ± 10.6
67f	−CF_3_	−H	−H	−OPr	524.49 ± 25.4	51.0 ± 7.33	524.49 ± 19.3	19.0 ± 6.01
67g	−CF_3_	−H	−H	−N (Me)_2_	550.27 ± 30.1	63.2 ± 4.81	550.27 ± 84.5	30.9 ± 7.53
67h	−CF_3_	−H	−OMe	−OH	34.84 ± 5.43	99.55 ± 1.23	140.14 ± 63.2	97.67 ± 3.26
67i	−CF_3_	−H	−OCH_2_O	−	38.53 ± 2.35	101.17 ± 5.83	77.42 ± 9.41	99.6 ± 13.5
67j	NO_2_	−H	−H	−H	4.93 ± 0.30	73.82 ± 5.37	35.1 ± 5.21	60.53 ± 5.58
67k	NO_2_	−OEt	−H	−H	3.71 ± 0.10	84.82 ± 3.73	15.0 ± 1.12	46.35 ± 7.85
67l	NO_2_	−OiPr	−H	−H	4.89 ± 0.29	80.71 ± 9.41	14.12 ± 1.05	31.69 ± 1.32
67m	NO_2_	−H	−OMe	−OH	1.81 ± 0.08	91.74 ± 2.35	19.49 ± 1.79	55.22 ± 8.85
67n	NO_2_	−H	−OMe	−OMe	2.5 ± 0.10	75.0 ± 9.35	301.9 ± 10.2	36.33 ± 6.20
67o	NO_2_	−OMe	−OMe	−OMe	3.23 ± 0.20	90.0 ± 4.56	43.65 ± 2.37	58.91 ± 7.81
Pimobendan					4.67 ± 0.83	93.22 ± 5.23	N.T	N.T
Carbachol					0.51 ± 1.9	106.3 ± 9.71	N.A	N.A
Nitrendipine					N.T	N.T	0.03 ± 0.003	98.90 ± 5.0

*N.T* Not tested, *N.A* Not active

**Scheme 27:**
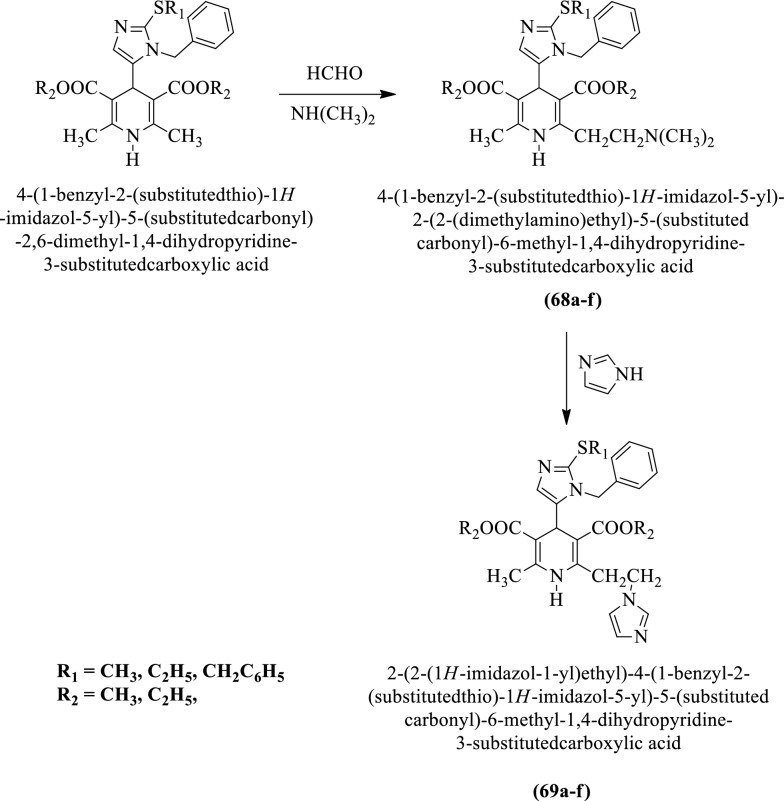
Synthesis of 2-(2-(1*H*-imidazol-1-yl)ethyl)-4-(1-benzyl-2-(substituted thio)-1H-imidazol-5-yl)-5-(substituted carbonyl)-6-methyl-1, 4-dihydropyridine-3-substituted carboxylic acid

Hadizadeh et al. [46] synthesized 2-(2-(1H-imidazol-1-yl)ethyl)-4-(1-benzyl-2-(substituted thio)-1H-imidazol-5-yl)-5-(substituted carbonyl)-6-methyl-1, 4-dihydropyridine-3-substituted carboxylic acid (Scheme 27) and evaluated for antihypertensive potential in rats and the results of anti-hypertensive activity were summarized in (Table 27, Hadizadeh et al. [46]).

**Table 27:** Antihypertensive activity of titled compounds (68a-f) in normotensive and hypertensive rats Hadizadeh et al. [46]

Compounds	MABP fall (SEM) in rats in doses C, in mg/kg b.w., i.v					
	Normotensive			Hypertensive		
	0.3	3	30	0.3	3	30
68a	26.00(2.00)	42.00(3.00)	47.20(3.03)	38.40(5.37)	46.40(2.19)	50.00(2.00)
68b	Nd	Nd	Nd	Nd	Nd	Nd
68c	22.00(2.00)	42.00(2.00)	57.2(2.16)	29.60(4.56)	54.40(7.79)	58.00(2.73)
68d	18.00(2.00)	42.00(2.00)	47.00(1.67)	22.40(3.58)	48.00(1.78)	49.20(1.78)
68e	Nd	Nd	Nd	Nd	Nd	Nd
68f	Nd	Nd	Nd	Nd	Nd	Nd
69a	17.20(2.68)	41.60(20.60)	53.20(2.28)	28.00(6.20)	52.80(11.79)	55.20(2.28)
69b	26.40(5.80)	37.20(1.55)	38.60(3.83)	29.00(2.9)	45.75(8.87)	50.80(6.60)
69c	23.20(7.69)	44.80(3.34)	56.80(3.34)	35.20(3.35)	56.00(4.00)	56.80(3.34)
69d	27.60(1.82)	37.40(1.15)	36.60(3.63)	29.00(5.10)	42.00(7.30)	44.5(7.60)
69e	15.40(0.27)	28.60(1.09)	33.00(1.41)	28.00(4.70)	36.80(1.60)	51.00(8.70)
69f	17.40(1.03)	26.60(3.19)	36.80(5.30)	24.80(4.56)	42.00(5.40)	48.00(7.40)
Nifedipine	27.20(2.68)	59.60(3.84)	Nd	42.40(5.36)	61.20(14.46)	Nd
DMSO	12.00(5.65)	12.00(3.65)	12.00(5.65)	14.80(6.72)	14.80(6.72)	14.80(6.72)

*MABP* Mean arterial blood pressure fall, *SEM* Standard error the mean are indicated in the parenthesis. All results were analyzed for statistically significant differences from control DMSO (0.3 mL/kg b.w., i.v) by analysis of variance and all showed significant difference. (*p* < 0.05), *Nd* not determined

**Scheme 28:**
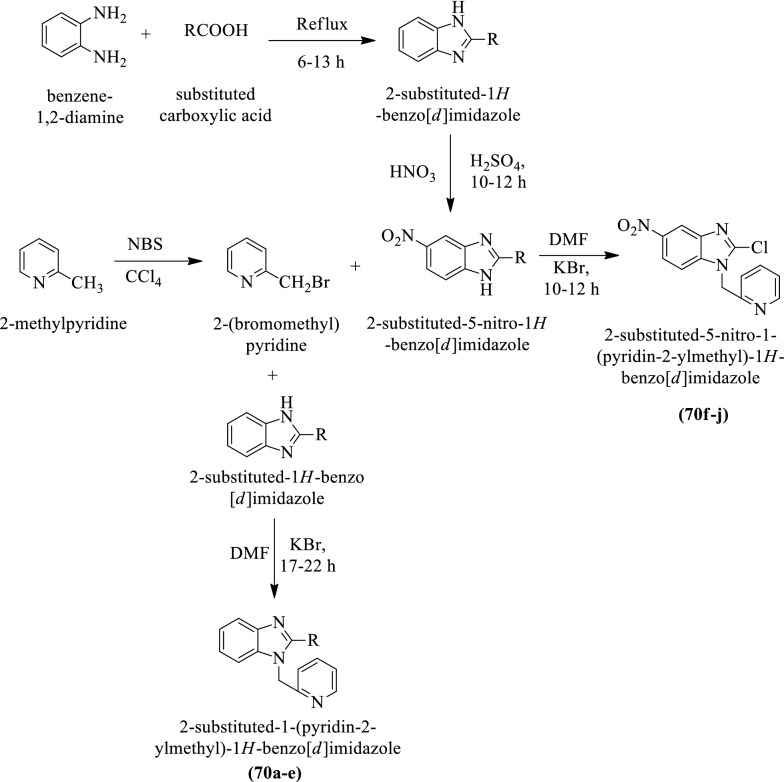
Synthesis substituted imidazole derivatives

Goyal et al. [22] synthesized 2-substituted-1-(pyridin-2-ylmethyl)-1H-benzo[d]imidazole derivatives (Scheme 28) and evaluated for antihypertensive potential and the results of activity were summarized in (Table 28, Goyal et al. [22]).

**Table 28:** Antihypertensive activity of the synthesized compounds (70a-j) Goyal et al. [22]

Haemodynamic parameters					
Compounds		SAP (mmHg)	DAP (mmHg)	MAP (mmHg)	HR (bpm)
70a	B	189 ± 7	129 ± 5	159 ± 6	311 ± 19
	A	161 ± 9*	105 ± 6*	121 ± 5*	298 ± 11
70b	B	189 ± 7	124 ± 8	154 ± 5	310 ± 18
	A	188 ± 6	122 ± 4	151 ± 7	320 ± 19
70c	B	206 ± 15	124 ± 8	151 ± 6	357 ± 15
	A	198 ± 18	119 ± 6	146 ± 5	337 ± 21
70d	B	217 ± 8	128 ± 6	160 ± 8	339 ± 17
	A	213 ± 7	132 ± 8	157 ± 9	330 ± 14
70e	B	221 ± 6	130 ± 5	157 ± 9	363 ± 16
	A	213 ± 3	129 ± 4	155 ± 8	347 ± 17
70f	B	178 ± 2	146 ± 7	151 ± 6	413 ± 28
	A	176 ± 3	144 ± 11	148 ± 9	402 ± 32
70g	B	194 ± 5	165 ± 8	180 ± 7	416 ± 18
	A	187 ± 7	155 ± 6	168 ± 6	409 ± 11
70h	B	158 ± 6	151 ± 9	155 ± 6	453 ± 29
	A	144 ± 5*	141 ± 8*	142 ± 9*	459 ± 21
70i	B	198 ± 7	154 ± 7	176 ± 7	410 ± 19
	A	197 ± 6	148 ± 6	183 ± 8	406 ± 14
70j	B	140 ± 6	118 ± 7	127 ± 5	511 ± 45
	A	138 ± 5	115 ± 4	125 ± 4	465 ± 28
Control	B	169 ± 6	145 ± 3	154 ± 6	415 ± 23
	A	168 ± 9	140 ± 4	149 ± 7	407 ± 29
Prazocin (3 mg/kg)	B	199 ± 7	156 ± 6	168 ± 6	418 ± 17
	A	176 ± 8*	138 ± 4*	141 ± 3*	411 ± 15

Haemodynamic effects shown on systolic blood pressure (SAP), Diastolic blood pressure (DAP), Mean arteriolar pressure (MAP) and Heart rate (HR) on SHRs treated with vehicle control and test compounds. Values were represented as mean ± SEM; n = 5; *p < 0.05

### Antitubercular activity

**Scheme 29:**
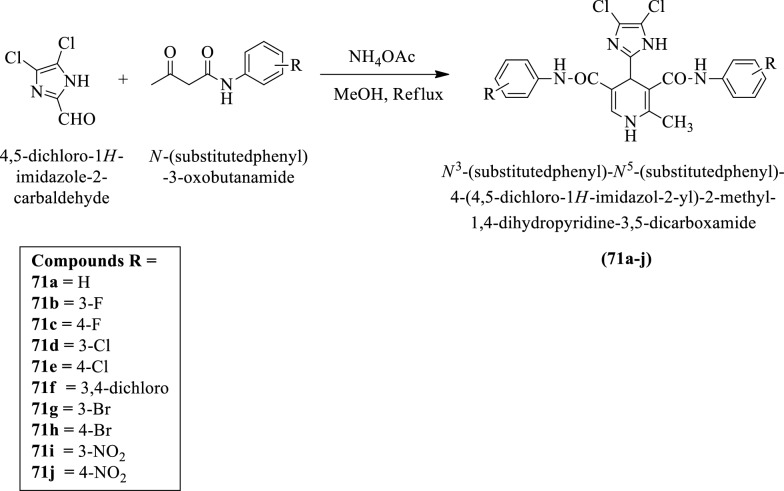
Synthesis of *N*^3^-(substituted phenyl)-*N*^5^-(substituted phenyl)-4-(4, 5-dichloro-1*H*-imidaVol-2-yl)-2-methyl-1, 4-dihydropyridine-3, 5-dicarboxamide

Amini et al. [47] synthesized N3-(substituted phenyl)-N5-(substituted phenyl)-4-(4,5-dichloro-1H-imidazol-2-yl)-2-methyl-1, 4-dihydropyridine-3,5-dicarboxamide (Scheme 29) and evaluated for anti-tubercular activity against *Mycobacterium tuberculosis* strain using rifampicin as reference drug. The conclusion of the anti-tubercular activity was presented in (Table 29, Amini et al. [47]).

**Table 29:** Antitubercular activity of the synthesized compounds (71a-j) against *Mycobacterium tuberculosis* (H_37_Rv strain) Amini et al. [47]

Compounds	R	Inhibition %
71a	H	9
71b	3-F	0
71c	4-F	13
71d	3-Cl	50
71e	4-Cl	12
71f	3,4-Cl_2_	34
71g	3-Br	1
71h	4-Br	0
71i	3-NO2	43
71j	4-NO_2_	43
Rifampicin		> 98

**Scheme 30:**
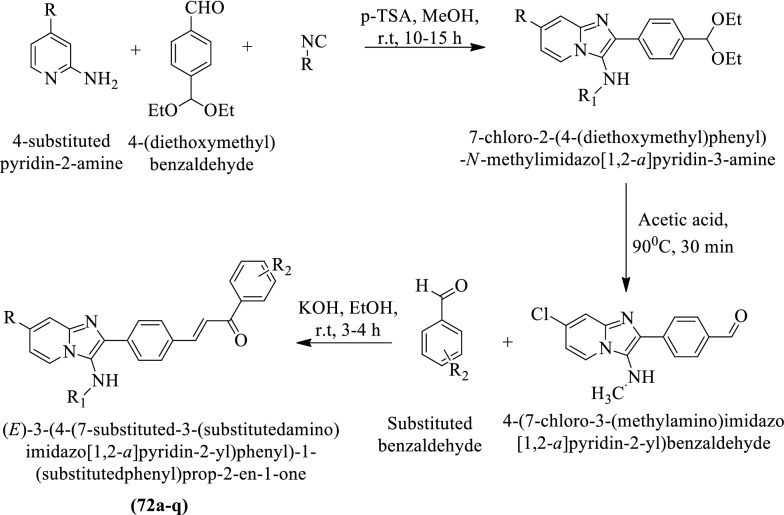
Synthesis of (*E*)-3-(4-(7-substituted-3-(substituted amino)imidazo[1,2-*a*] pyridin-2-yl)phenyl)-1-(substituted phenyl)prop-2-en-1-one

Pandey et al. [48] synthesized (E)-3-(4-(7-substituted-3-(substituted amino)imidazo[1,2-a] pyridin-2-yl)phenyl)-1-(substituted phenyl)prop-2-en-1-one (Scheme 30) and evaluated for anti-tubercular potential against *Mycobacterium tuberculosis* strain by **MB 7H10** agar medium using Ethambutol and Pyrazinamide as a reference drug. The conclusion of the activity was presented in (Table 30, Pandey et al. [48]).

**Table 30:**
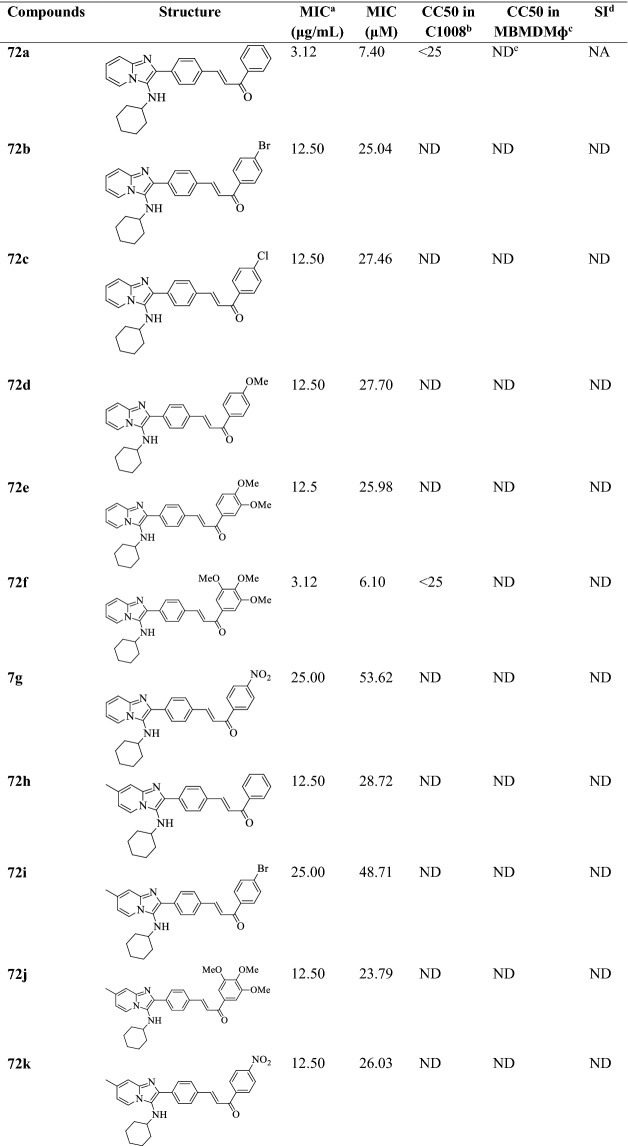

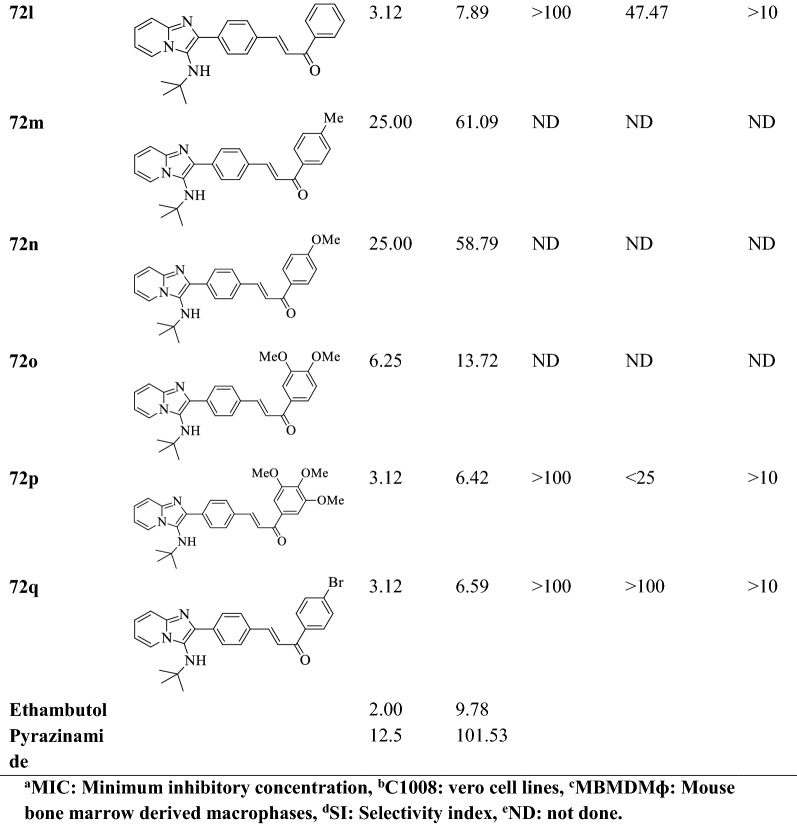
Antitubercular activity of synthesized compounds (72a-q) against *M. tuberculosis* H_37_Rv Pandey et al. [48]

**Scheme 31:**
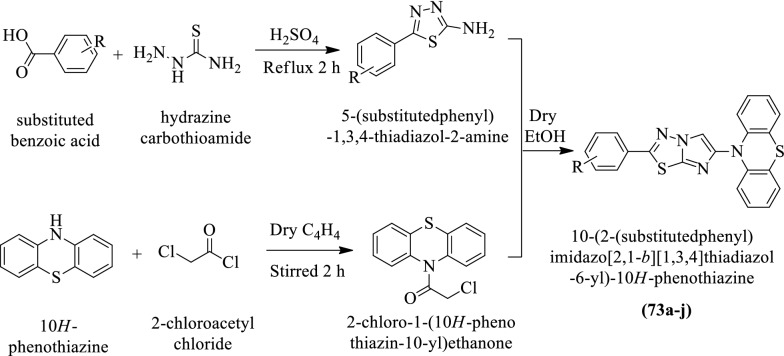
Synthesis of 10-(2-(substituted phenyl)imidazo[2,1-*b*][1, 3, 4]thiadiazol-6-yl)-10H-phenothiazine

Makwane et al. [49] synthesized 10-(2-(substituted phenyl)imidazo[2,1-b][1,3,4]thiadiazol-6-yl)-10H-phenothiazine by using (Scheme 31) and evaluated for antitubercular activity by **(L.J)** agar method against *Mycobacterium tuberculosis* **H**_**37**_**Rv** strain using Isoniazid as reference drug and MIC values of these derivatives were calculated. The conclusion of anti-tubercular activity was presented in (Table 31, Makwane et al. [49]).

**Table 31.** Antitubercular activity of the synthesized compounds (73a-j) Makwane et al. [49]

Compounds	Ar1	Antitubercular activity inhibition (%) (ppm) M. tuberculosis H37Rv strain		Antitubercular activity MIC* (μg/mL) M. tuberculosis H37Rv strain
		25	50	
73a	C_6_H_5_	22	45	12
73b	2-ClC_6_H_4_	32	79	7.5
73c	3-ClC_6_H_4_	36	80	6.5
73d	4-ClC_6_H_4_	32	78	7
73e	2-BrC_6_H_4_	29	73	10
73f	3-BrC_6_H_4_	30	76	8.5
73g	4-BrC_6_H_4_	30	75	9
73h	2-NO_2_C_6_H_4_	28	82	5.5
73i	3- NO_2_C_6_H_4_	27	84	4
73j	4-NO_2_C_6_H_4_	32	83	5

**Scheme 32:**
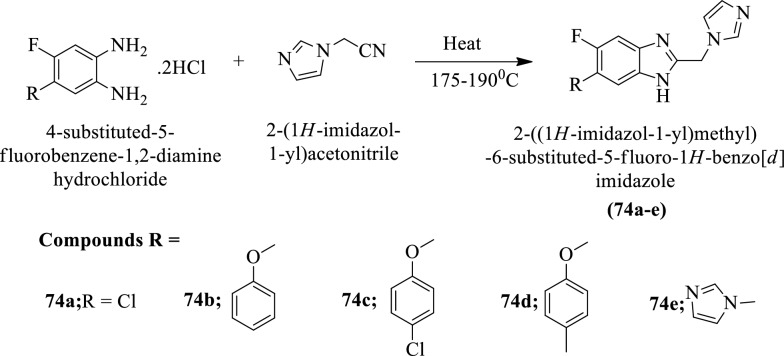
Synthesis of 2-((1*H*-imidazol-1-yl)methyl)-6-substituted-5-fluoro-1*H*-benzo[*d*]imidazole

Nandha et al. [23] synthesized 2-((1H-imidazol-1-yl)methyl)-6-substituted-5-fluoro-1H-benzo[d]imidazole (Scheme 32) and evaluated for anti-tubercular activity against *Mycobacterium tuberculosis* strain by **MABA** assay using Isoniazid as a reference drug. The conclusion of anti-tubercular activity was presented in (Table 32, Nandha et al. [23]).

**Table 32:** Antitubercular activity of synthesized derivatives (74a-e) against *M. tuberculosis* H_37_Rv strain Nandha et al. [23]

Compounds	MIC (μg/mL) MABA
74a	100
74b	50
74c	25
74d	50
74e	12.5
Isoniazid	0.78

*MIC* Minimum inhibitory concentration, *MABA* Microplate Alamar Blue Assay (visual)

**Scheme 33:**
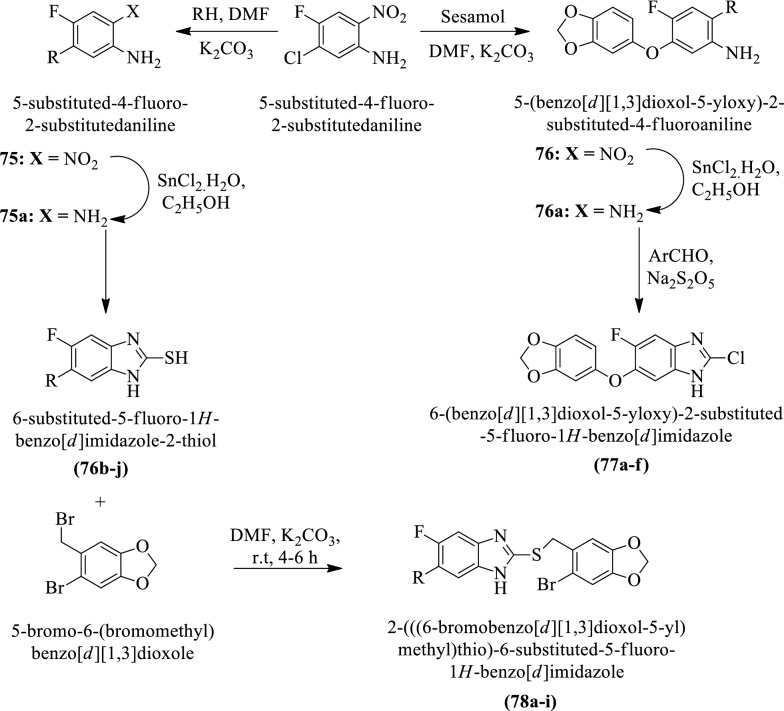
Synthesis of 6-(benzo[*d*][1, 3]dioxol-5-yloxy)-2-substituted-5-fluoro-1*H*-benzo[*d*] imidazole and 2-(((6-bromobenzo[*d*][1,3]dioxol-5-yl)methyl)thio)-6-substituted-5-fluoro-1 h-benzo[*d*]imidazole

Nandha et al. [50] synthesized 6-(benzo[d][1,3]dioxol-5-yloxy)-2-substituted-5-fluoro-1H-benzo[d] imidazole (Scheme 33) and evaluated for anti-tubercular activity against *Mycobacterium tuberculosis* **(ATCC27294)** by **MABA** assay using streptomycin, ciprofloxacin, and pyrazinamide as a reference drug. The conclusion of the activity was presented in (Table 33, Nandha et al. [50]).

**Table 33:**
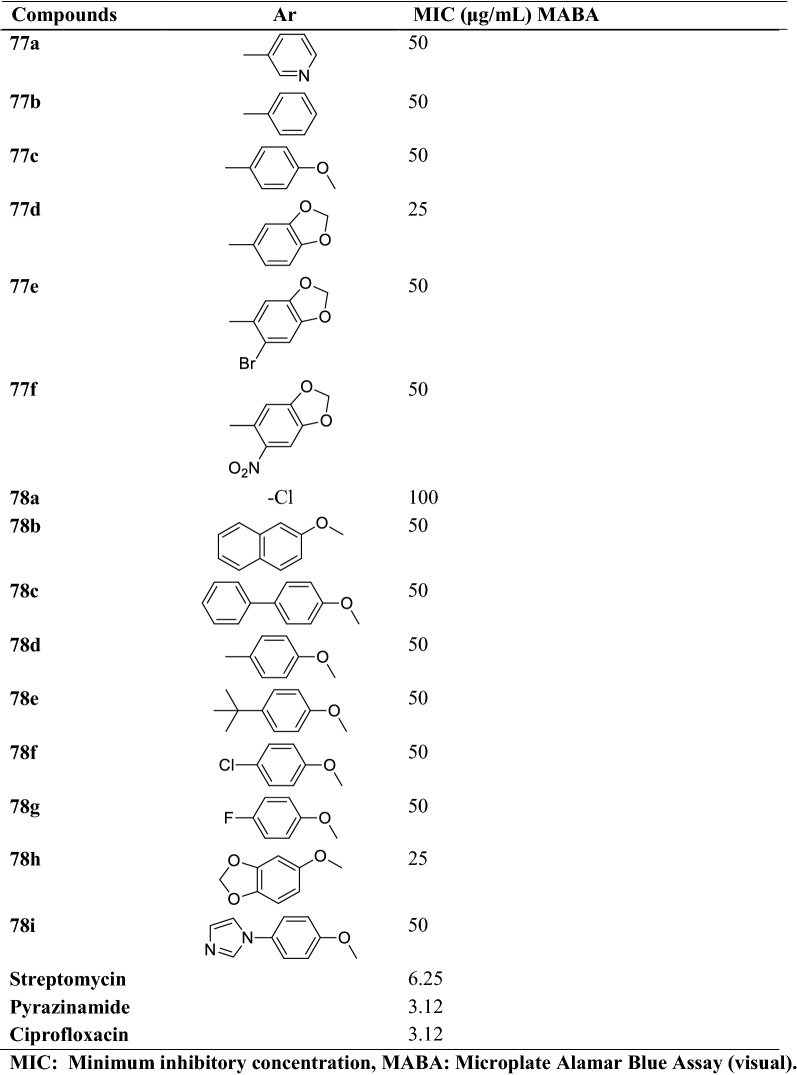
Antitubercular activity of synthesized derivatives (77a-f) and (78a-i) Nandha et al. [50]

**Scheme 34:**
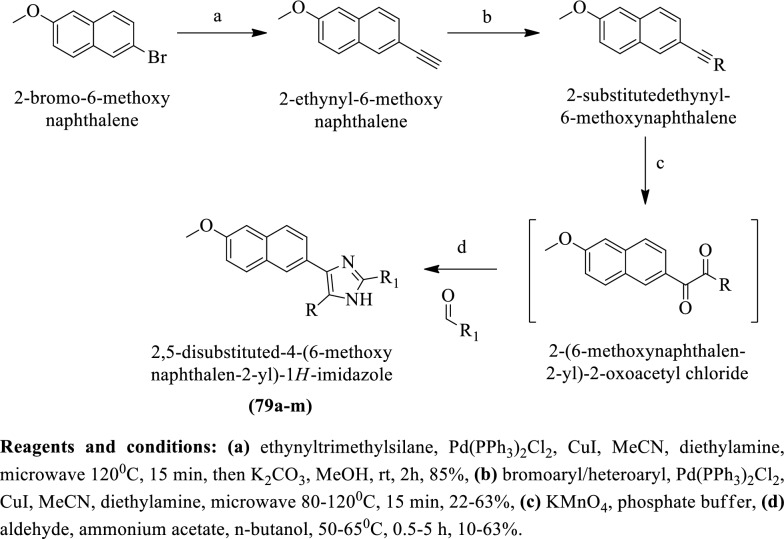
Synthesis of 2,5-disubstituted-4-(6-methoxynaphthalen-2-yl)-1*H*-imidazole

Gising et al. [51] synthesized 2,5-disubstituted-4-(6-methoxynaphthalen-2-yl)-1H-imidazole by using (Scheme 34). The anti-tubercular potential of these derivatives was evaluated against *Mycobacterium tuberculosis* strain and MIC values of these derivatives were calculated. The conclusion of anti-tubercular activity was presented in (Table 34, Gising et al. [51]).

**Table 34:**
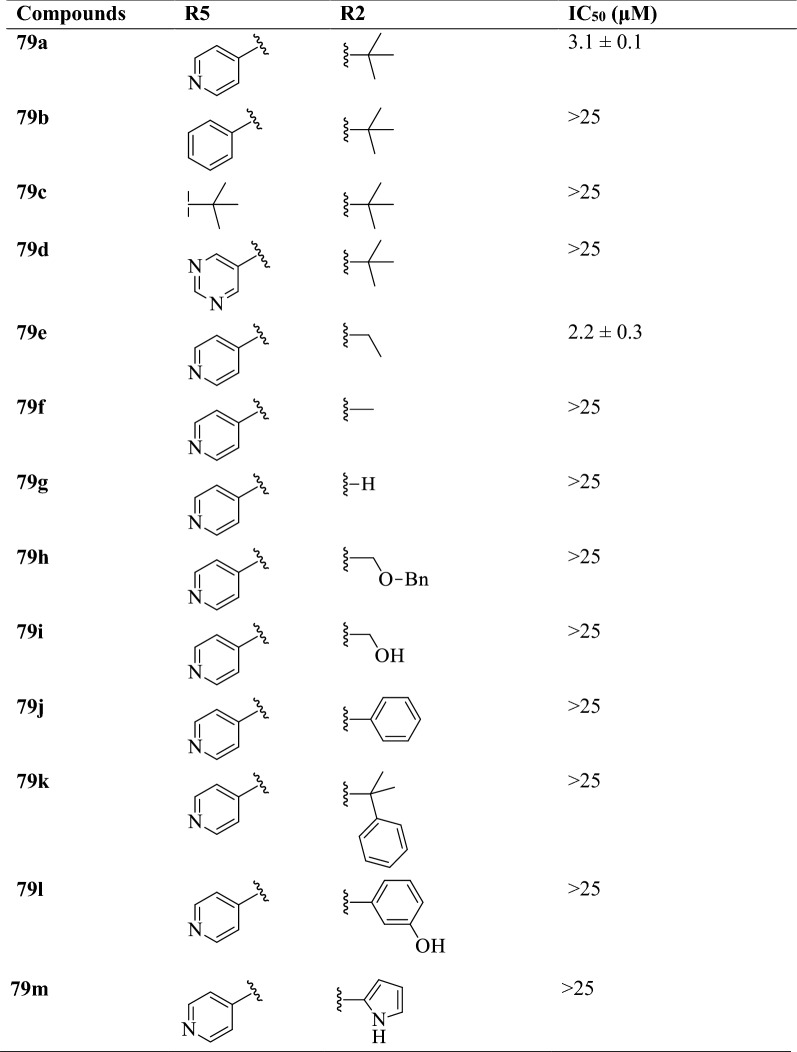
Antitubercular activity of synthesized derivatives (79a-m) Gising et al. [51]

**Scheme 35:**
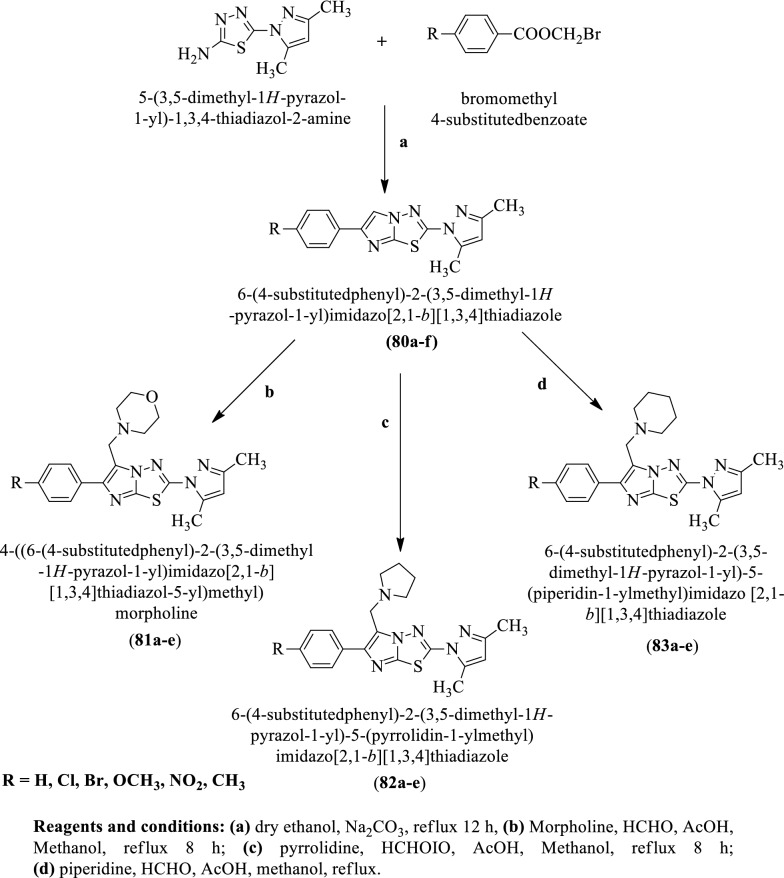
Synthesis of substituted phenyl imidazole derivatives

**Syed et al. **[52] synthesized 6-(4-substituted phenyl)-2-(3,5-dimethyl-1H-pyrazol-1-yl)imidazo [2,1-b][1,3,4] thiadiazole (Scheme 35) and evaluated for anti-tubercular potential against *Mycobacterium tuberculosis* strain. Compounds **80a, 80b, 81a, 82a**, and **83a** showed the most potent anti-tubercular activity as compared to others. The conclusion of anti-tubercular activity was presented in (Table 35, Syed et al. [52]).

**Table 35:** Antitubercular activity of synthesized compounds (80a-83e) Syed et al. [52]

Compounds	MIC (μg/mL) MABA
80a	10
80b	10
81a	10
81b	25
82a	10
82b	25
83a	10
83b	25
83c	25
83e	25
Streptomycin	7.5

**Scheme 36:**
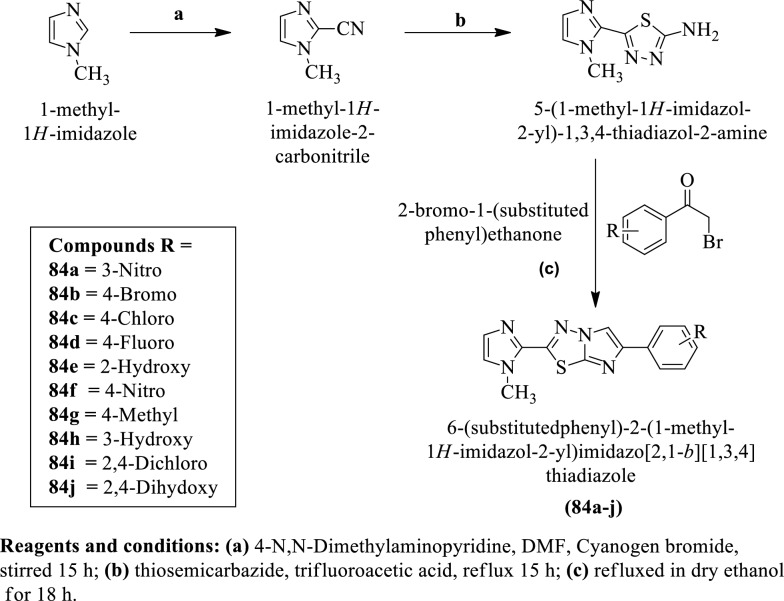
Synthesis of 6-(substituted phenyl)-2-(1-methyl-1H-imidazol-2-yl) imidazo [2,1-b] [1,3,4] thiadiazole

Patel et al. [53] synthesized 6-(substituted phenyl)-2-(1-methyl-1H-imidazol-2-yl) imidazo [2,1-b] [1,3,4] thiadiazole (Scheme 36) and evaluated for anti-tubercular activity against *Mycobacterium tuberculosis* and MIC values of these derivatives were calculated. The conclusion of anti-tubercular activity was presented in (Table 36, Patel et al. [53]).

**Table 36:** Antitubercular activity of synthesized compounds (84a-j) Patel et al. [53]

Compounds	R	Inhibition %	Activity	MIC (μg/mL)	IC_50_	SI
84a	3-Nitro	91	+	4.34	10.56	2.43
84b	4-Bromo	94	+	5.78	11.4	1.97
84c	4-Chloro	95	+	5.48	12.3	2.24
84d	4-Fluoro	90	+	4.86	8.5	1.74
84e	H	16	−	> 6.25	–	–
84f	4-Nitro	98	+	3.14	9.8	3.12
84 g	4-Methyl	18	−	> 6.25	–	–
84 h	3-Methyl	30	−	> 6.25	–	–
84i	2,4-Dichloro	92	+	5.66	10.3	1.81
84j	2,4-Dihydroxy	−	−	> 6.25	–	–
Rifampicin				0.125–0.25		> 10

**Scheme 37:**
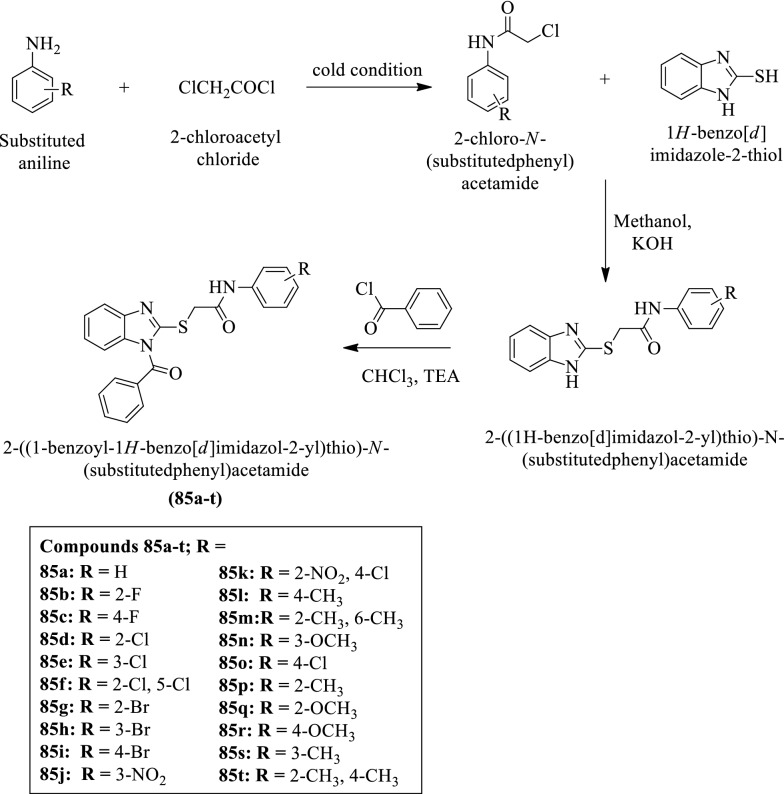
Synthesis of 2-((1-benzoyl-1H-benzo[*d*]imidazol-2-yl) thio)-N-(substituted phenyl) acetamide

Yadav et al. [54] synthesized 2-((1-benzoyl-1H-benzo[d]imidazol-2-yl) thio)-N-(substituted phenyl) acetamide (Scheme 37) and evaluated for anti-tubercular activity against *Mycobacterium tuberculosis* strain and MIC values of these derivatives were calculated. Streptomycin was used as a reference drug and the results of anti-tubercular activity were presented in (Table 37, Yadav et al. [54]).

**Table 37:** Antitubercular activity of synthesized compounds (85a-t) Yadav et al. [54]

Compounds	Diameter of zone of inhibition (mm) against H37Rv (NCFT/TB/537)	MIC (μg/mL)	MLC (μg/mL)
85a	> 20	12.5	25
85b	> 20	12.5	25
85c	> 20	12.5	25
85d	> 20	12.5	25
85e	08	17.8	28.12
85f	> 20	12.5	25
85 g	10	15	28
85 h	> 20	12.5	25
85i	08	17.8	28.12
85j	20	12.5	25
85 k	10	15	28
85 l	> 20	12.5	25
85 m	> 20	12.5	25
85n	NA	NA	NA
85o	> 20	12.5	25
85p	10	15	28
85q	> 20	12.5	25
85r	> 20	12.5	25
85 s	NA	NA	NA
85t	10	15	28
Streptomycin	> 20	12.5	25

## Conclusion

In this present review article, we have summarized different pharmacological activities of 1,3-diazole containing compounds. From this study, we have found that 1,3-diazole containing compounds can be synthesized by various kinds of synthetic routes, and these derivatives having a wide range of biological activities such as antitumor, antitubercular, antimicrobial, antihypertensive and antioxidant, etc. This review article established the fact that 1,3-diazole act as useful templates for further modification or derivatization to design more potent biologically active compounds.

## Data Availability

All data are provided in the manuscript or cited in the references.

## Abbreviations

AMR: Antimicrobial resistance
DNA: Deoxyribonucleic acid
DMF: Dimethylformamide
TEBA: Triethyl benzyl ammonium chloride
MTT: 3-(4, 5-Dimethylthiazol-2-yl)-2,5-diphenyltetrazolium bromide
C-A4: Combretastatin-A4
SRB: Sulforhodamine B assay
DLA: Dalton’s Lymphoma Ascites cell line
EAC: Ehrlich’s ascites carcinoma cell lines
DPPH: 2,2-Diphenyl-1-picrylhydrazyl
FRAP: Ferric reducing ability of plasma
SHR: Spontaneously hypertensive rats
MB: Middle brook
MABA: Microplate Alamar blue assay
L.J: Lowenstein-Jensen
IC_50_: Half maximal inhibitory concentration
HeLa: Henrietta Lacks
TEA: Triethanolamine
DMSO: Dimethyl sulphoxide
MIC: Minimum inhibitory concentration
TBAB: Tetrabutylammonium bromide
NCFT: National Centre of Fungal Taxonomy
MLC: Minimum Lethal concentration
p-TSA: P-Toluenesulfonic acid
MW: Microwave
CAN: Cerric ammonium nitrate
(4-SB) T (4-SPh)PHSO4: (4-Sulfobutyl)tris(4-sulfophenyl) phosphonium hydrogen sulfate
